# Associations of cannabis use disorder with cognition, brain structure, and brain function in African Americans

**DOI:** 10.1002/hbm.25324

**Published:** 2020-12-19

**Authors:** Marinka M. G. Koenis, Joke Durnez, Amanda L. Rodrigue, Samuel R. Mathias, Aaron F. Alexander‐Bloch, Jennifer A. Barrett, Gaelle E. Doucet, Sophia Frangou, Emma E. M. Knowles, Josephine Mollon, Dominique Denbow, Katrina Aberizk, Molly Zatony, Ronald J. Janssen, Joanne E. Curran, John Blangero, Russell A. Poldrack, Godfrey D. Pearlson, David C. Glahn

**Affiliations:** ^1^ Department of Psychiatry School of Medicine, Yale University New Haven Connecticut USA; ^2^ Olin Neuropsychiatry Research Center Institute of Living Hartford Connecticut USA; ^3^ Department of Psychology Stanford University Stanford California USA; ^4^ Department of Psychiatry Boston Children's Hospital & Harvard Medical School Boston Massachusetts USA; ^5^ Department of Psychiatry Icahn School of Medicine at Mount Sinai New York New York USA; ^6^ Department of Human Genetics, and South Texas Diabetes and Obesity Institute School of Medicine, University of Texas Rio Grande Valley Brownsville Texas USA; ^7^ Department of Neuroscience Yale University New Haven Connecticut USA

**Keywords:** cognition, DTI, marijuana, morphometry, resting state, white matter

## Abstract

Although previous studies have highlighted associations of cannabis use with cognition and brain morphometry, critical questions remain with regard to the association between cannabis use and brain structural and functional connectivity. In a cross‐sectional community sample of 205 African Americans (age 18–70) we tested for associations of cannabis use disorder (CUD, *n* = 57) with multi‐domain cognitive measures and structural, diffusion, and resting state brain‐imaging phenotypes. Post hoc model evidence was computed with Bayes factors (BF) and posterior probabilities of association (PPA) to account for multiple testing. General cognitive functioning, verbal intelligence, verbal memory, working memory, and motor speed were lower in the CUD group compared with non‐users (*p* < .011; 1.9 < BF < 3,217). CUD was associated with altered functional connectivity in a network comprising the motor‐hand region in the superior parietal gyri and the anterior insula (*p* < .04). These differences were not explained by alcohol, other drug use, or education. No associations with CUD were observed in cortical thickness, cortical surface area, subcortical or cerebellar volumes (0.12 < BF < 1.5), or graph‐theoretical metrics of resting state connectivity (PPA < 0.01). In a large sample collected irrespective of cannabis used to minimize recruitment bias, we confirm the literature on poorer cognitive functioning in CUD, and an absence of volumetric brain differences between CUD and non‐CUD. We did not find evidence for or against a disruption of structural connectivity, whereas we did find localized resting state functional dysconnectivity in CUD. There was sufficient proof, however, that organization of functional connectivity as determined via graph metrics does not differ between CUD and non‐user group.

## Graphical abstract

In a large sample collected irrespective of cannabis used to minimize recruitment bias, we confirm the literature on poorer cognitive functioning in cannabis use disorder (CUD), and an absence of volumetric brain differences between CUD and non‐CUD. A disruption of structural connectivity remains equivocal. We find localized resting state functional dysconnectivity in CUD, and sufficient proof that organization of functional connectivity as determined via graph metrics does not differ between CUD and non‐users.

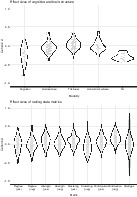

## 1 INTRODUCTION

Views on cannabis use are changing in the United States. After legalization of medical cannabis and more recent decriminalization/legalization of recreational use in many states, it is likely that more people will use cannabis (Pearlson, 2020). Despite a recent meta‐analysis reporting no link between legalization of medical cannabis and increased use (Sarvet et al., 2018), cannabis use and cannabis use disorder (CUD) are increasing (Compton, Han, Jones, Blanco, & Hughes, 2016; Hasin et al., 2017; Wang, Davies, Halmo, Sass, & Mistry, 2018), whereas the perceived risk of cannabis use is decreasing (Compton et al., 2016; Wen, Hockenberry, & Druss, 2019). A critical issue is that the evidence base regarding the safety of cannabis use is incomplete. Specifically, more information is needed regarding potential risks of long‐term cannabis use, particularly on brain function.

The importance of examining the effect of cannabis on brain phenotypes is underscored by evidence that chronic use is associated with lower global neuropsychological function and underperformance in tasks of verbal learning and memory, attention, and psychomotor function (Broyd, van Hell, Beale, Yücel, & Solowij, 2016; Crane, Schuster, Fusar‐Poli, & Gonzalez, 2013; Scott et al., 2018). Although this association may be bidirectional (Jackson et al., 2016; Meier et al., 2018) the cognitive effects of cannabis are particularly relevant for adolescents and young adults (Volkow, Baler, Compton, & Weiss, 2014). Furthermore, effects are more prominent within 72 hr of last use with limited evidence that effects persist after longer periods of abstinence (Scott, Slomiak, et al., 2018).

Initial findings on brain morphometry mostly report a decreased hippocampal and amygdala volume (Lorenzetti, Solowij, & Yücel, 2016; Murray et al., 2017; Rocchetti et al., 2013), but these have largely been superseded by recent large‐scale studies that did not identify cannabis effects on cortical thickness or subcortical volumes (Chye et al., 2019; Gillespie et al., 2018; Meier, Schriber, Beardslee, Hanson, & Pardini, 2019; J. M. Orr, Paschall, & Banich, 2016; Scott et al., 2018; Thayer et al., 2017). Nevertheless, a smaller hippocampal volume has been associated with current cannabis dependence (Chye et al., 2019), and tetrahydrocannabinol (THC, a psychoactive component of cannabis) positive urine but not lifetime use (Owens, Sweet, & MacKillop, 2020). In contrast, white matter integrity, as inferred from fractional anisotropy (FA) derived from diffusion tensor imaging (DTI) may be reduced in cannabis users (Arnone et al., 2008; Becker, Collins, Lim, Muetzel, & Luciana, 2015; Epstein & Kumra, 2015; Gruber, Dahlgren, Sagar, Gönenç, & Lukas, 2014; Jacobus, Squeglia, Bava, & Tapert, 2013; Jakabek, Yücel, Lorenzetti, & Solowij, 2016; Manza, Yuan, Shokri‐Kojori, Tomasi, & Volkow, 2019; Murray et al., 2017; J. M. Orr et al., 2016; Shollenbarger, Price, Wieser, & Lisdahl, 2015), even after months of abstinence (Ashtari, Cervellione, Cottone, Ardekani, & Kumra, 2009). Although many studies have looked at functional connectivity and cannabis use, most studies involve activation tasks and/or region of interest approach (Blest‐Hopley, Giampietro, & Bhattacharyya, 2018). Indeed, only a handful of studies have looked at resting state connectivity using a whole brain approach. These studies report higher and lower functional connectivity in different regions, probably due to small sample sizes and different methodological approaches (Cheng et al., 2014; Filbey, Gohel, Prashad, & Biswal, 2018; Orr et al., 2013; Thijssen et al., 2017).

While inconsistencies in the literature could reflect a number of methodological differences, three issues appear to be limiting our understanding of the relationship between cannabis use and brain structure and function. First, many studies use small sample sizes with limited statistical power, which reduces the chance of detecting a true effect and decreases the likelihood that a statistically significant result reflects a true effect (Button et al., 2013). Second, the definition of cannabis use varies dramatically between studies, potentially limiting generalizability. Third, ascertainment strategies can result in subtle biases that can skew results, particularly for case–control studies where cases and controls are drawn from different populations (Delgado‐Rodriguez & Llorca, 2004; Kopec & Esdaile, 1990). To overcome these limitations, we studied the association of CUD on cognitive and neuroimaging measures in a relatively large sample (*n* = 205) of individuals recruited without regard to their cannabis use status and used DSM criteria to define CUD. Additionally, the use of multimodal imaging and detailed cognitive assessment enables the simultaneous investigation of the effect of CUD on multiple brain and behavioral variables in the same individuals.

We hypothesized that CUD would be associated with abnormal structural and functional connectivity involving brain regions that are rich in cannabis receptors, including cerebellum, subcortical structures, cingulate, and frontal areas (Burns et al., 2007; Glass, Faull, & Dragunow, 1997). We also expected to replicate prior findings on poorer cognitive function in verbal tasks and negative findings regarding the associations between cannabis use and brain morphometry.

## 2 MATERIALS AND METHODS

### 2.1 Participants

Participants (aged 18–70 years) were recruited via flyers, advertisements in local newspapers, and Craigslist as the control group of an ongoing study into psychosis (Mathias et al., 2017). To maintain generalizability, participants were allowed to have common psychiatric disorders (except for psychosis) and rates of psychiatric disorders in our sample reflect national averages in this population (Tables 1 and S1a–d). All participants underwent formal diagnostic assessments using the Structured Clinical interview for DSM‐IV (First, Spitzer, Gibbon, & Williams, 2002) (see Supporting Information). Exclusion criteria included a history of major medical disorders, severe head injury, MRI contraindication, IQ < 70, dementia, traces of drugs other than THC in urine, and drug intoxication during cognitive or MRI assessment.

**TABLE 1:** Sample characteristics

	Non‐CUD	CUD	*p*
*N* ^a^	148	57	
Male, *n* (%)	62 (41.9%)	28 (49.1%)	.432^e^
Age (range)	40 (18–70)	37 (19–69)	.182^f^
Duration of CB use, mean (range in years)^b^	‐	11 (1–40)	
Education, median (range)^c^	4 (2–8)	3 (2–6)	<.001^a^
On social disability, *n* (%)	17 (11.5%)	3 (5.3%)	.292^b^
Axis I psychiatric diagnoses, *n* (%)			
No diagnosis	95 (64.19%)	‐	
Depression	14 (9.46%)	8 (14.04%)	.327^e^
PTSD	12 (8.11%)	4 (7.02%)	1.000^e^
Anxiety disorders	6 (4.05%)	5 (8.77%)	.184^b^
ADHD	0 (0%)	1 (1.75%)	.278^e^
Alcohol abuse/dependence	30 (20.27%)	21 (36.84%)	.019^e^
Drug other than CB abuse/dependence	15 (10.14%)	20 (35.09%)	.019^e^
Medication, *n* (%)^d^			
Anxiolytic	3 (1.99%)	0 (0%)	.562^e^
Antidepressant	6 (3.97%)	2 (3.51%)	1.000^e^
Atypical antipsychotic^d^	2 (1.32%)	0 (0%)	1.000^e^
Anticonvulsant^d^	2 (1.32%)	0 (0%)	1.000^e^
Alcohol and nicotine			
Nr drinks per month, mean (SD)	9 (19)	14 (17)	.001^f^
Currently smoking, *n* (%)	46 (31.08)	42 (73.68)	< .001^e^
FTND, median (range)^b^	0 (0–9)	3 (0–9)	< .001^g^

*Note:* Diagnoses are lifetime diagnoses.

Abbreviations: CB, cannabis; FTND, Fagerström test for nicotine dependence (ranges from 0 to 12).

^a^ Wilcoxon rank sum test.

^b^ Fisher's exac*t* test.

^c^ N_cognition_ = 142 + 53; N_anatomy_ = 107 + 45; N_DTI_ = 110 + 42; N_resting_state_ = 92 + 39 non‐CUD + CUD respectively; see Table S1a–d.

^d^ missing data cannabis duration (*n* = 3); FTND (*n* = 36, equal percentage in the non‐CUD and CUD group),

^e^ ordinal: (1) Grade 6 or less; (2) Grade 7–12 without graduation; (3) high school/GED Graduate; (4) part college; (5) 2‐year college/trade school graduate; (6) 4‐year college graduate; (7) part graduate school or professional school; (8) complete graduate or professional school.

^f^ None of the participants used mood stabilizers or lithium. Atypical antipsychotic medication was used for treatment of MDD; Anticonvulsant was used for nerve pain.

^g^ Welch's *t* test.

Participants were diagnosed with CUD (*n* = 57) if they (a) met DSM‐IV criteria for current or partially remitted CUD (*n* = 7 for abuse, *n* = 23 for current dependence, *n* = 11 for partial remission); or (b) met criteria for remitted CUD for >1 year but had positive THC urine test (*n* = 9) or mentioned they used socially (*n* = 6). Individuals who did not use were defined as non‐CUD (*n* = 148). Non‐CUD participants were excluded if they tested positive for THC (*n* = 13) or mentioned that they used occasionally (*n* = 18) or use was unknown (*n* = 1). Figure S1 provides an overview of final sample size after quality control for each modality. Since (supervised) abstinence of 3 to 28 days normalizes functional connectivity (Blanco‐Hinojo et al., 2017; Jacobus et al., 2012), cannabinoid receptor density (D'Souza et al., 2016; Hirvonen et al., 2012), and cognitive performance (Scott, Slomiak, et al., 2018), individuals in full remission were classified as past‐CUD and placed in a separate group. This group was too small (*n* = 22) for analyses and was excluded from the current study. The protocol was approved by institutional review boards at Hartford Hospital and Yale University. All participants provided written informed consent.

### 2.2 Cognitive assessment

Under supervision, participants completed a cognitive test battery (“Charlie,” https://github.com/sammosummo/Charlie, (Mathias et al., 2017)), which included fully computerized and computer‐aided administration of tests listed in Table 2. A composite score of general intellectual functioning denoted by *g* was derived as the first PCA component (see Supporting Information). Final group sizes were 142 non‐CUD, 53 CUD.

**TABLE 2:** Included tests from cognitive tests battery

Domain	Test	Description
General cognitive ability	*g*	First component of principal component analyses using all cognitive measures listed below.
Verbal intelligence	Wechsler test of adult Reading WASI vocabulary	Subjects read aloud a list of 50 irregularly spelled words. Score is the number of correctly pronounced words. Subjects answer questions about the meaning of words (e.g., what does winter mean?).
Abstract reasoning	WASI matrix	Subjects view an incomplete matrix and select the response that completes the matrix.
Verbal and semantic fluency	COWAT: Controlled Oral word association test	Subjects list as many words as possible in 60 s. In the first three trials, the words must begin with the letters, F, A, and S (verbal fluency). In the fourth trial, the words must be animals (semantic fluency).
Processing speed and executive function	Trail making A and B	Computerized Trail making: Subjects click on circles presented on the screen in a specified order. The order is either consecutive letters (A), or alternating numbers and letters (B). Output is total time taken to complete the trail.
Processing speed	Digit symbol	A key of digit symbol pairs is presented at the top of the screen and subjects indicate whether a target digit symbol pair presented at the center of the screen matches any pair from the key. Subjects complete as many trials as possible in 90 s.
Verbal memory	CVLT: California verbal learning test	Subjects hear an audio recording of 16 words and repeat out loud as many words as they can recall. This is repeated for five trials. Sum correct is the total number of correct recalls over all five trails. On the sixth trial (recall condition), subjects list as many words as they can recall without first hearing the audio recording. In the recognition condition, subjects recognize the 16 target words presented alongside 32 non‐target words.
Working memory	Span forward and backward Letter number sequencing	Subjects hear sequences of letters or numbers that increase in length throughout the trials and repeat these sequences out loud. In the forward condition, subjects repeat the sequences in the order in which they heard them. In the backward condition, subjects repeat the sequences in reverse order. Same as above, but the sequences contain both letters and numbers and subjects repeat the letters in numerical order, followed by the letters in alphabetical order.
Facial memory	Penn face memory test	Subjects see images of faces and are asked to try to remember them. After they have seen all the faces, they perform a recognition‐memory task. Each trial comprises a face (either an old face or a new one), and subjects make old/new judgments. Direct and delayed correct responses were averaged.
Emotional memory	Penn emotion recognition test	Subjects see a color image of a face expressing an emotion (happy, sad, fearful, and angry) or with a neutral expression. Subjects make their responses by clicking on the words printed to the screen. There is no feedback and there are no practice trials.
Spatial memory	Corsi SCAP: Spatial capacity delayed response test	Subjects observe the sequence of blocks in which circles appear, and then repeat the sequence back in order. The task starts with a small number of blocks and gradually increases in length up to nine blocks. Three different conditions (clicking the block where circles appeared (order irrelevant), clicking in order (similar to the original Corsi), clicking blocks in order where circles appeared, then click in order the blocks were crosses appeared) were averaged to obtain one score. On each trial, the subject sees a study array comprising three to five yellow circles in random positions on the screen. The study array is removed and, after a delay, is replaced by a single green circle (the probe). The subject indicates whether the probe has the same spatial location as one of the original circles. In this version of the SCAP, there are 14 three‐, 14 four‐ and 14 five‐item trials.
Sustained attention	IPCPTs: Identical pairs continuous performance test	On each trial, the subject sees a three‐item symbol array, and presses the space bar each time the current array matches the array from the previous trial (effectively a 1‐back task). Trials have a duration of 1.5 s. There are 200 trials in the test phase.
Motor speed	Orientation test	Subject sees either a blue square (during the first 10 trials) or a blue square and a red circle (during the last 10 trials) positioned randomly on the screen. The task is to click on the blue square as quickly as possible. After each trial the blue square becomes smaller and the red square becomes larger. It is similar to the mouse practice task from Gur et al., 2001. Output is total time taken to complete the test.

### 2.3 Neuroimaging

Imaging was conducted on a Siemens Skyra 3 T scanner at the Olin Neuropsychiatry Research Center, Institute of Living, Hartford Hospital. Sequences were modeled on the Human Connectome Project (HCP) imaging protocols (Glasser et al., 2016; Smith et al., 2013; Sotiropoulos et al., 2013). Anatomical scans were acquired with a T1‐weighted gradient echo pulse sequence with the following parameters: voxel size = 0.8 mm isotropic; TE/TR/TI = 2.09/2400/1010 ms; flip angle = 8°; duration = 7:02 min. DTI scans with 90 directions and b‐values of 0 and 2000 s/mm^2^ (voxel size = 1.8 mm isotropic; TE/TR = 92.80/4250 ms; duration = 6:50 m) were acquired in 4 scans: two left‐to‐right and two right‐to‐left phase encoding direction. Resting‐state data included four 5:08 min scans (two left‐to‐right and two right‐to‐left phase encoding direction) where participants were asked to keep their eyes open and let their minds wander freely. Acquisition parameters were: TE/TR = 36/720 ms; flip angle = 52°; voxel size = 2.1 mm isotropic.

T1‐weighted images were analyzed with Freesurfer version 5.3 (Fischl, 2012) to obtain cortical thickness and surface area measurements for 68 cortical and volumetric measures for 14 subcortical regions and the cerebellum of the Desikan‐Killiany parcellation (Desikan et al., 2006). Image quality was assessed via MRIQC (Esteban et al., 2017), excluding scans with MRIQC score ≥ 0.5. Final group sizes were 107 non‐CUD, 45 CUD.

Diffusion images were processed using FMRIB's Software Library (FSL) (Smith et al., 2004) version 10. Preprocessing included brain extraction, correction for motion and eddy current distortions, and tensor fitting resulting in individual FA maps. FA maps were fed into Tract‐Based Spatial Statistics (TBSS) (Smith et al., 2006). Average FA was calculated for the whole skeleton and 20 white matter tracts based on the John's Hopkins University white matter atlas (Hua et al., 2008). Two motion estimates calculated during the DTI distortion correction were used as covariates. Final group sizes were 110 non‐CUD, 42 CUD.

Preprocessing of resting‐state data was based on HCP pipelines (Glasser et al., 2013). Framewise displacement (FD) and root‐mean‐square change in blood‐oxygen‐level‐dependent (BOLD) signal from volume to volume (DVARS) was calculated and timepoints with FD > 0.5 mm were removed. Runs with >20% high motion time points were discarded. Most subjects (67%) had all 4 runs (in both groups). Final group sizes were 92 non‐CUD, 39 CUD. See Supporting Information for details on preprocessing.

The cortex was parcellated according to the Gordon atlas (Gordon et al., 2016), subcortical regions were segmented with the Harvard‐Oxford atlas (Desikan et al., 2006), and cerebellum with FSL's probabilistic atlas (Diedrichsen, Balsters, Flavell, Cussans, & Ramnani, 2009). Total number of brain parcels was 382. Global signal regression was applied (based on all voxels) and pair‐wise correlations between all 382 regions were Fisher r‐to‐z transformed. The correlation matrix was checked for outliers. First, edge‐wise outliers (>4 *SD*) were detected. Second, for each subject the number of outlier‐edges was calculated. Based on the histogram of the number of outlier‐edges per subject, five subjects (two CUD, three non‐CUD) where defined as outliers (>1,000 outlier‐edges). These subjects did not deviate in their mean connectivity. Analyses were repeated without these subjects.

### 2.4 Organization of functional connectivity

The organizational structure of resting‐state functional connectivity (FC) was assessed with two complementary approaches: network‐based static (NBS) and graph‐theoretical metrics.

NBS (Zalesky, Fornito, & Bullmore, 2010) is a nonparametric method that exploits the extent to which connections comprising the contrast of interest (determined with an F‐test to allow for two‐sided effects) are interconnected. The size of the connected component is based on the number of connections (extent) or sum of the F‐statistics of all edges (intensity).

Graph‐theoretical metrics were estimated using the Brain Connectivity Toolbox (BCT, www.brain-connectivity-toolbox.net). We focused on metrics of (a) global connectivity (global efficiency); (b) regional connectivity (degree, strength, and clustering coefficient); and (c) modular organization (participation coefficient, modularity).

Because graph metrics are sensitive to the density of the graph (i.e., the fraction of present connections to all possible connections), we selected the same number of edges for each participant, namely the top 1 to 40% (increments of 1%) of the strongest positive or negative edges. This upper bound was based on the density of positive versus negative edges in each participant, thus ensuring that the top 40% of positive edges included the same number of positive only edges for each participant (and likewise for top 40% negative edges). Graphs based on positive and negative correlations were analyzed separately for global efficiency, clustering coefficient, and strength. Modularity and participation coefficient were computed on the union of positive‐ and negative‐correlation based graphs (thus, graph density for these was twice that of other metrics). A Louvain approach with negative asymmetry was used to estimate modularity (Rubinov & Sporns, 2011).

Graph‐theoretical metrics were computed for each density threshold (1–40%), thus forming a curve as a function of density. The difference between the groups was computed as the area between their respective curves. Permutations (*n* = 50,000) of group membership were used to assess significance. When regional output was created (clustering coefficient, strength, degree, participation coefficient), *p*‐values were corrected for multiple testing (382 regions) with an FDR‐correction. Effect sizes and Bayes factors were computed over the mean over all densities.

### 2.5 Statistical analyses

Covariates common to all analyses were age, age^2^, sex, and their interactions. Cognitive, morphometry, and DTI data were visually checked for normality (density and Q‐Q plots) and transformed if necessary (only for cognitive data, with either data^*e*^; data^−1^; log[data], depending on the distribution). In the structural data, outliers (>4 *SD*) were removed as they likely reflect segmentation errors. Differences between groups were assessed via *t* tests, and multiple regression (glm function) in R (R Core Team, 2017). The threshold for statistical significance was set at *p* < .05 after FDR correction. Bayes factors (BF) (Jarosz & Wiley, 2014; Jeffreys, 1961) were computed on imputed data with BayesFactor package in R (Morey & Rouder, 2018) against a null‐interval of (−0.1, 0.1) (Morey & Rouder, 2011). BF > 3 is considered substantial, BF > 10 is strong evidence for the alternative, and BF < 0.3 is substantial evidence for the null (Jeffreys, 1961). Bayes Factors with intermediate values (i.e., greater than 0.3 but less than 3) neither confirm nor reject a specific hypothesis. Rather, such factors are considered inconclusive, without enough evidence to definitively determine the presence or absence of an effect. The posterior probability of association (PPA) was used to interpret BF with multiple testing (Stephens & Balding, 2009) and can be interpreted as PPA < 0.25 for substantial evidence and PPA < 0.10 for strong evidence for the null hypothesis (see Supporting Information).

Post hoc analyses examined the relationship of (log‐transformed) duration of cannabis use and age of onset of cannabis use on measures that significantly differed between groups. We tested for the influence of confounding variables by regressing out (in separate analyses) the effects of number of alcoholic drinks/month, lifetime diagnosis of alcohol use disorder (AUD), lifetime diagnosis of substance use disorder other than cannabis or nicotine (SUD), and years of education approximated as an ordinal variable (see Supporting Information), and social disability status as a proxy for social economic status (although this did not differ between groups, *p* = .292). Because correlations between nicotine use and brain structure (Liao, Tang, Liu, Chen, & Hao, 2012) and function (Filbey et al., 2018) have been reported, we also regressed out the effects of nicotine dependence (Fagerström test) for any significant brain metrics.

## 3 RESULTS

### 3.1 Cognition

**FIGURE 1:**
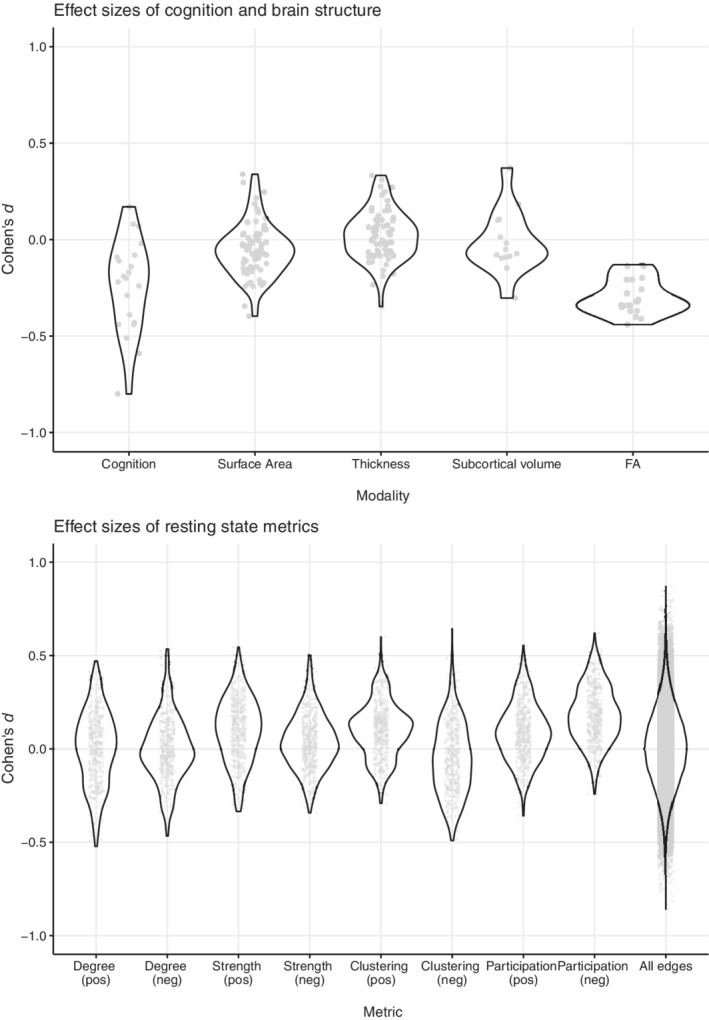
Overview of all effect sizes for all modalities examined in this study; each dot represents one test or region. Negative effect sizes mean variable of interest is “lower” in CUD group. Effect sizes of modularity and positive/negative efficiency were − 0.02, 0.29, 0.15, respectively

Seven tests showed FDR‐corrected group differences in the domains of general cognitive ability, verbal intelligence, verbal memory, working memory and motor speed (Cohen's *d =* −0.39 to −0.80, *p* = <.011, BF > =1.9, Figure 1, Table 3). As cognitive test‐scores are often correlated (Figure S2), we performed a multiple regression analysis including each of the test scores (excluding *g*; variance inflation factors <2.67), to determine if a single measure was differentially associated with CUD status. Only the total sum of correctly recalled words significantly differed between the CUD and non‐CUD group (*p* = .0006) in this analysis.

**TABLE 3:** Means (*SD*) of cognitive tests for subjects with and without cannabis use disorder (CUD)

Domain	Test	Non‐CUD	CUD	Cohen's *d*	*p*	BF
General cognitive ability	*g*	0.12 (1.03)	−0.31 (0.83)	−0.44	**.003** ^a^	3.71
Verbal and semantic fluency	COWAT, animal	0.02 (1.00)	−0.06 (1.01)	−0.09	.591	0.13
	COWAT, F‐A‐S	0.04 (1.00)	−0.10 (1.00)	−0.14	.404	0.17
Verbal intelligence	WTAR	0.15 (1.05)	−0.42 (0.71)	−0.59	**<.0001**	37.91
	Vocabulary	0.14 (1.04)	−0.36 (0.78)	−0.51	**.0004**	11.72
Verbal memory	CVLT, sum correct	0.20 (0.98)	−0.55 (0.83)	−0.80	**<.0001** ^b^	3,216.71
	CVLT, recall	0.11 (0.99)	−0.31 (0.97)	−0.43	**.009** ^a^	3.26
	CVLT, recognition	−0.02 (1.08)	0.05 (0.74)	0.07	.616	0.12
Spatial memory	Corsi	0.06 (0.99)	−0.16 (1.02)	−0.22	.216	0.30
	SCAP	0.03 (1.00)	−0.08 (1.00)	−0.11	.481	0.15
Emotional memory	Emotion recognition	0.00 (0.94)	−0.01 (1.17)	−0.02	.920	0.11
Facial memory	Face memory	0.02 (1.03)	−0.06 (0.93)	−0.08	.615	0.12
Working memory	Forward	0.05 (0.99)	−0.15 (1.02)	−0.20	.226	0.27
	Backward	0.08 (1.02)	−0.21 (0.94)	−0.29	.069	0.61
	Letter‐number	0.10 (1.03)	−0.28 (0.87)	−0.39	**.011** ^a^	1.89
Sustained attention	IPCPTs	0.05 (1.00)	−0.14 (0.99)	−0.19	.251	0.25
Abstract reasoning	Matrix reasoning	0.07 (1.05)	−0.18 (0.82)	−0.24	.095	0.39
Motor speed	Orientation time	0.12 (1.01)	−0.32 (0.91)	−0.44	**.005** ^a^	3.67
Processing speed	Digit symbol coding	0.05 (0.99)	−0.13 (1.02)	−0.17	.296	0.22
Processing speed & executive function	Trail making A	−0.02 (1.02)	0.06 (0.96)	0.08	.627	0.12
	Trail making B	−0.05 (0.99)	0.12 (1.01)	0.17	.301	0.22

*Note:* Values are standardized residuals. Bold indicates significant at *p* < .05, FDR corrected.

Abbreviations: BF, Bayes factor; COWAT, controlled oral word association test; CVLT, California verbal learning test; IPCPT, identical pairs continuous performance test; SCAP, spatial capacity delayed response test; WTAR, Wechsler adult reading test.

^a^ Significant at *p* < .05 (uncorrected) after controlling for lifetime substance abuse/dependence other than cannabis. The other tasks remain significant at *p* < .05 FDR corrected.

^b^ Significant at *p* < .05 (FDR corrected) after controlling for education. The other tasks are no longer significant.

### 3.2 Brain morphometry

No statistically significant neuroanatomical differences were observed in subcortical volumes, cerebellar volume, cortical thickness or cortical surface‐area (Cohen's *d* = −0.40 to 0.37; *p* > .026 uncorrected, BF < 1.5; Figure 1, Tables S2 and S3). BF was <0.3 for the majority of the regions, including many frontal and subcortical regions, providing support for the null hypothesis (Jeffreys, 1961). Although findings were inconclusive (0.3 < BF < 1.5) for 22 regions, these regions did not differ between the groups when correcting for the number of tests (PPA < 0.14 when BF < 1.5 and prior probability <0.1).

### 3.3 Diffusion imaging

Mean FA of several bundles was lower the CUD group (Cohen's *d* < −0.41, *p <* .05 uncorrected; Figure 1; Table S4). However, none were statistically significant after FDR correction. BFs ranged from 1.07–2.19, indicating inconclusive evidence for a group difference in FA of these bundles.

### 3.4 Resting‐state connectivity

#### 3.4.1 Network‐based statistics

**FIGURE 2:**
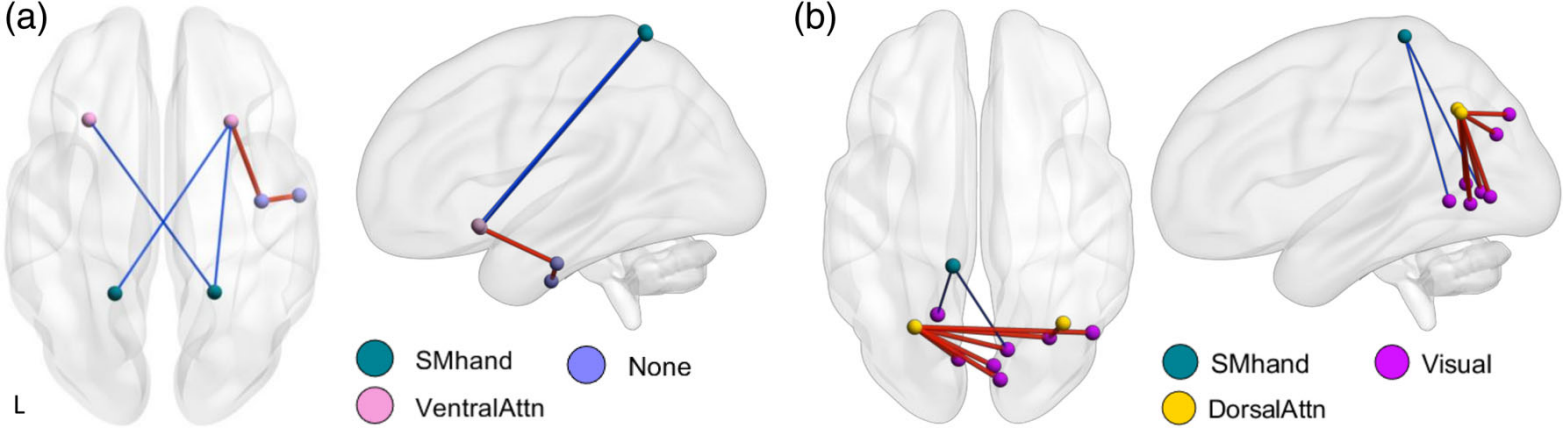
Affected network in CUD at F ≥ 15, based on intensity (a) and extent (b). Red/thick edge, stronger functional connectivity in CUD; Blue/thin edge, weaker functional connectivity in CUD. SM, somatomotor cortex; Attn, attention

Significant components based on intensity were found after thresholding for group differences at F ≥ 15, 16 and 17 (*p* = .034, .023, and .018, respectively). The intensity‐component at F ≥ 15 included 5 edges connecting 6 nodes: the bilateral motor‐hand area in the superior parietal gyrus (SPG), bilateral insula, right fusiform gyrus, and the right inferior temporal gyrus. In CUD, FC was lower between SPG and insular regions; but higher between the right insula and fusiform gyrus, and between the fusiform gyrus and the inferior temporal gyrus (Figures 2 and S3, Table 4). Based on extent, one component was found at F ≥ 15 (*p* = .030) comprising 9 edges over 10 mostly occipital nodes. FC between the bilateral middle occipital gyrus and nodes of the visual network was higher in CUD, whereas FC between the left precuneus and the bilateral lingual gyrus was lower (Figures 2 and S3, Table 4).

**TABLE 4:** Mean (*SD*) functional connectivity and effect sizes of edges in the NBS component (intensity, F ≥ 15) and FDR significant edges

	Non‐CUD	CUD	Cohen's *d*	*p*	BF^a^
NBS—intensity					
R fusiform to R Inf temporal	−0.020 (0.082)	0.046 (0.099)	0.750	.00059^c^ ^d^ ^e^ ^f^ ^g^ ^h^	106
R sup parietal to L insula	0.023 (0.094)	−0.043 (0.048)	−0.793	.00000^c^ ^d^ ^g^	214
R fusiform R to R insula	−0.015 (0.074)	0.043 (0.088)	0.750	.00053^c^ ^d^ ^e^ ^f^ ^g^	107
R sup parietal to R insula^b^	0.022 (0.068)	−0.051 (0.066)	−1.078	.00000^c^ ^g^	180,522
L sup parietal to R insula	0.021 (0.082)	−0.051 (0.088)	−0.863	.00004^c^ ^g^	728
NBS—extent					
L mid occipital to L lingual	−0.022 (0.084)	0.048 (0.115)	0.747	.00104^c^ ^d^ ^e^ ^f^ ^g^ ^h^ ^i^	101
L precuneus to L lingual	0.015 (0.096)	−0.060 (0.102)	−0.756	.00025^c^ ^d^ ^f^ ^g^ ^h^ ^i^	117
L mid occipital to R mid temporal	−0.035 (0.128)	0.087 (0.175)	0.851	.00023^c^ ^d^ ^f^ ^g^ ^h^ ^i^	584
L mid occipital to R fusiform	−0.039 (0.132)	0.080 (0.183)	0.801	.00053^c^ ^d^ ^f^ ^g^ ^h^ ^i^	244
R mid occipital to R fusiform	−0.041 (0.152)	0.092 (0.218)	0.763	.00105^c^ ^d^ ^f^ ^g^ ^h^ ^i^	132
L mid occipital to R cuneus	−0.028 (0.107)	0.065 (0.135)	0.798	.00034^c^ ^d^ ^f^ ^g^ ^h^ ^i^	235
L mid occipital to R lingual	−0.026 (0.089)	0.055 (0.138)	0.767	.00132^c^ ^d^ ^f^ ^g^ ^h^ ^i^	141
L precuneus to R lingual	0.017 (0.089)	−0.051 (0.098)	−0.745	.00039^c^ ^d^ ^f^ ^g^ ^h^ ^i^	99
L mid occipital to cuneus	−0.027 (0.100)	0.059 (0.126)	0.786	.00040^c^ ^d^ ^f^ ^g^ ^h^ ^i^	191
FDR					
L accumbens to R hippocampus	−0.019 (0.068)	0.037 (0.072)	0.822	.00008^c^ ^g^	351
L mid frontal to vermis crus I	0.019 (0.070)	−0.041 (0.075)	−0.837	.00006^f^	457
R sup parietal to R insula^b^	0.022 (0.068)	−0.051 (0.066)	−1.078	.00000^g^	180,522

*Note:* Values are standardized residuals.

Abbreviations: BF = Bayes factor.

^a^ See Supporting information on interpretation of BF and multiple testing.

^b^ This edge is present in the FDR and NBS‐intensity network.

^c^ Not significant after controlling for nicotine dependence.

^d^ Not significant after controlling for drinks/month.

^e^ Not significant after controlling for SUD.

^f^ Not significant after controlling for education.

^g^ Not significant when excluding outliers.

^h^ Not significant after controlling for DVARS.

^i^ Not significant after controlling for lifetime AUD.

Of the entire FC matrix, three edges reached FDR‐corrected significance. One edge overlapped with the NBS‐intensity component (right SPG to right insula; Table 4, Figure S4). This was also the only edge that had sufficient evidence for a group difference (PPA > 0.95).

#### 3.4.2 Graph‐theoretical metrics

There were no group differences in global or regional metrics. There were no group differences in mean FC between resting state modules; mean FC strength within module or mean FC strength between each module and all other modules; or mean FC over the entire matrix. See Figure 1 for effect sizes, Table S5 for an overview of marginal findings at *p* < .005 uncorrected. Although BF > 6, PPA < 0.01 indicates strong evidence that none of the metrics differed between the groups.

#### 3.4.3 Global signal regression

To test for bias related to the global signal, we reran the FC analyses on data without global signal regression (GSR). There were no group differences in the NBS approach. Preparing the data for the graph metrics, it was noticed that the CUD‐group had stronger mean FC over the entire matrix (*p* = .0363, Cohen's d = 0.40), though evidence was inconclusive (BF = 1.3). For the GSR processed matrix there was evidence for the absence of a group difference in mean FC (*p* = .297, BF = 0.26). Group differences of FC between each of the resting state modules (120 combinations) did not reach FDR corrected significance (*p* > .0024, BF < 13.5, PPA <0.66). There was no evidence for or against a group difference in within network FC (*p* < .032, BF < 1.42, PPA < 0.41) nor mean outward FC of each network (*p* < .0091, BF < 3.74, PPA < 0.65). See Supporting Information for details.

Percentage of positive and negative edges was more variable in the non‐GSR data; in order to set the number of edges equal across all participants, positive graph metrics were computed at 1–25% density, and negative metrics at 1–13% density (as opposed to 40% for both positive and negative metrics in the main GSR analyses). There were several group differences at *p* < .005 uncorrected (Table S6). However, as in the GSR analyses, none of the findings survived FDR correction and PPA suggested an absence of a group difference (PPA < 0.20 for 24 of 28 variables, the remaining 4 were inconclusive PPA < 0.33). Findings did not overlap with results done on GSR data with the same density range (Table S7).

### 3.5 Post hoc analyses

#### 3.5.1 Duration of use

Significant results mentioned above were tested for correlation with duration of cannabis use and age of first cannabis use (in CUD group only). None of the cognitive or resting state variables correlated with these metrics, although evidence is inconclusive (0.2 < BF <1.8; see Supporting Information).

#### 3.5.2 Cross modality correlations

Cognitive and neuroimaging measures found to differ between the CUD and non‐CUD group were correlated to examine potential common effects across cognitive and brain measures. None of these correlations survived FDR correction and BFs were inconclusive at best (BF < 9.8, PPA < 0.15; see Supporting Information).

#### 3.5.3 Confounding variables

Analyses showing significant results were repeated after regressing out the effects of confounding variables to determine if results were influenced by comorbidity.

Group differences in cognition remained significant after adjusting for alcoholic drinks/month and AUD, but four tests did not reach FDR significance after covarying for SUD (Table 3). When correcting for education, only verbal learning reached (FDR‐corrected) significance. Correcting for social disability status did not change the results.

The NBS‐intensity components were roughly the same when alcoholic drinks/month, AUD, SUD, education, social disability, or DVARS were added as covariates. The connections between the SPG and right insula were the most stable. The NBS‐intensity component did not reach significance when corrected for nicotine dependence, and showed a different network when excluding five outliers. The NBS‐extent component remained significant only after correction for SUD and social disability. Edges found at FDR significance were stable after controlling for above mentioned confounding variables. See Table 4.

## 4 DISCUSSION

We investigated the association between CUD and measures of cognition, brain structure, and brain function in a large sample selected without regard to cannabis use status. Consistent with prior studies, CUD individuals showed poorer performance on measures of verbal intelligence, verbal memory, working memory, and motor speed. We did not find conclusive evidence for lower FA in the CUD group. Functional connectivity (FC) was altered in a component that included lower FC between the bilateral superior parietal gyrus (SPG) and right insula. In contrast, there was substantial evidence that neither neuroanatomical measures nor did graph‐theoretical metrics of FC differed between groups. Results were not related to alcohol drinking behavior, AUD, or SUD, although nicotine dependence explained a portion of the variance in FC.

Apart from acute effects (Ranganathan et al., 2017; Schuster et al., 2018), the causal link between cannabis use and educational attainment (Defoe, Khurana, Betancourt, Hurt, & Romer, 2018; Lynskey & Hall, 2000), or cognition is unclear (Morin et al., 2019; Scott, Slomiak, et al., 2018). A study on a cannabinoid receptor 1 (CB1) antagonist suggests that altered CB1‐signaling is involved in acute THC‐induced verbal memory impairment (Englund et al., 2016). Frequent cannabis use is associated with a downregulation of these receptors, but receptor density returns to baseline after abstinence (D'Souza et al., 2016; Hirvonen et al., 2012). Similarly, cognitive impairments (Scott, Slomiak, et al., 2018; Tait, Mackinnon, & Christensen, 2011) and FC (Blanco‐Hinojo et al., 2017) are recovered after abstinence, suggesting a causative role. However, longitudinal twin studies show that individuals at risk for substance abuse could have cognitive vulnerabilities before onset of cannabis use (Jackson et al., 2016; Meier et al., 2018; Ross et al., 2019), although there is also evidence from a longitudinal study for cognitive effects after persistent use (Meier et al., 2012). When correcting for education, many of the cognitive and FA differences between CUD and non‐CUD disappeared, except for verbal memory and FC. Verbal memory is one of the most robustly found domains to be affected in relation to cannabis use (Broyd et al., 2016; Crane et al., 2013). Possibly, these metrics may be associated with CUD through a different pathway.

The absence of significant morphometric differences associated with CUD is consistent with previous large studies on recreational cannabis use (Orr et al., 2016; Scott, Rosen, et al., 2018; Thayer et al., 2017), with similar effect sizes (Scott, Rosen, et al., 2018; Weiland et al., 2015). Our findings add to the literature that morphometric differences are absent even when individuals who meet CUD diagnostic criteria are compared with non‐users from the same population. However, a few recent large studies did find morphometric differences in association with cannabis use. Chye, Lorenzetti, et al. (2019); Chye, Suo, et al., 2019) found smaller hippocampi in CUD compared with controls and compared with nondependent users, but not in nondependent users compared with controls. Owens et al. (2020) found smaller hippocampi in THC+ participants, but not in participants with lifetime CUD, whereas Manza et al. (2019) found lower cortical thickness and gray matter density in the precuneus in lifetime CUD compared with carefully matched controls. These differences in results and sample characteristics suggest that different phenotypes (e.g., THC+, current vs. lifetime CUD, frequency of usage, etc.) may have different associations with brain morphometry. In addition, it emphasizes the need for replication studies.

Although the literature is consistent in reporting lower structural connectivity in participants with CUD (Arnone et al., 2008; Ashtari et al., 2009; Becker et al., 2015; Epstein & Kumra, 2015; Gruber et al., 2014; Jacobus et al., 2009; Jakabek et al., 2016; Manza et al., 2019; Murray et al., 2017; Orr et al., 2016; Shollenbarger et al., 2015; Zalesky et al., 2012), we did not find conclusive evidence for lower FA in the CUD group (0.3 < BF < 3). Functional connectivity studies, however, are more inconsistent. Studies using a whole brain approach to investigate CUD‐related resting state FC (Cheng et al., 2014; Filbey et al., 2018; C. Orr et al., 2013; Thijssen et al., 2017) have reported that cannabis users have: higher fractional amplitude of low‐frequency fluctuations in right superior frontal gyrus, SPG, semilunar node of the cerebellum, and weaker interhemispheric connectivity in the medial frontal cortex and pyramid of the cerebellum (C. Orr et al., 2013); stronger connectivity between frontal and precentral gyri, and between frontal and cingulate gyri (Cheng et al., 2014); weaker connectivity in the salience network and posterior cingulate gyrus (Filbey et al., 2018). The largest study (130 CUD, 47 controls) found no cannabis‐use associated differences in intra‐network connectivity but did report a negative association between duration of cannabis use and connectivity of the executive control network with the auditory and a sensorimotor network in cannabis users (Thijssen et al., 2017). We did not find differences in inter‐network connectivity of data processed with global signal regression (GSR); in non‐GSR data there could be (evidence was inconclusive) *stronger* inter‐network FC between the cingulo‐parietal (CP) and auditory network, and between the CP and somato‐motor network in CUD. These inconsistent results, emphasized by the difference between results with and without GSR, illustrate the need for more standardized methodological approaches and resist drawing generalized conclusions.

We report weaker FC between bilateral motor‐hand regions in the SPG and the bilateral insula in the NBS‐intensity and FDR approach, even after correcting for several confounders. In contrast, the NBS‐extent finding of stronger FC between nodes in the occipital cortex reached only marginal significance and did not survive correction for confounding variables. Although without GSR there were no group differences in NBS networks, the global signal (as inferred from mean FC over the entire matrix) was higher in the CUD group. Our results suggest that after removal of this signal, the CUD group still had different FC between specific regions. It should be stressed that the multiple comparison correction of NBS is at the component‐level rather than the edge‐level, meaning that the null hypothesis is not rejected for any specific edge, only for the component as a whole. The edges between the right SPG and right insula which reach FDR significance are not subject to this limitation. However, these findings were attenuated when controlling for nicotine dependence. A study on nicotine addiction reported that weaker FC between the insula (seed region) and pre‐ and postcentral gyri was related to higher chances of relapse (Addicott, Sweitzer, Froeliger, Rose, & McClernon, 2015). Although it is likely that these connections are not unique to nicotine addiction but substance abuse related (c.f. the involvement of the insula in drug craving [Naqvi, Gaznick, Tranel, & Bechara, 2014]), our results might be influenced by the higher prevalence of nicotine smokers in the CUD group. Taking that into account in combination with the marginal significance, our FC results should be treated with caution and replication is needed.

We did not find conclusive evidence for the presence or absence of a correlation between age of onset and duration of use and cognition or FC. The literature is also inconclusive on this association as some studies report that earlier onset and longer use have a more detrimental effect on cognition and the brain, whereas other studies report an absence of such association. For a comprehensive overview we refer the reader to (Nader & Sanchez, 2018). Discrepancies in the literature could be due to the definition of “cannabis users,” the fidelity of self‐report (Prince, Conner, & Pearson, 2018), or that early‐onset users may have a higher intensity and frequency of use compared with late‐onset users (Gruber, Sagar, Dahlgren, Racine, & Lukas, 2012). Additionally, THC content in cannabis preparations has increased from 3 to 12% between 1980 and 2014 (ElSohly et al., 2000; ElSohly et al., 2016). Although users may self‐titrate their desired level of THC consumption (Bidwell et al., 2018), this makes the cumulative THC intake difficult to estimate and compare. Last, specific timing of high frequency and/or high potent cannabis use may have a differential effect on outcomes. Frequency and potency of use could differ while age of onset is the same; more frequent use during adolescence versus (early) adulthood is likely to have more detrimental effects on cognition and the brain (Volkow et al., 2014, but see Meier et al., 2019) given that adolescence is a vulnerable period of educational opportunities and brain maturation. Future research should attempt to take these dynamics of cannabis use into account.

This study has several limitations. First, causality cannot be inferred from cross‐sectional studies. Second, we lack detailed information such as days since last use and intensity of recent use (with e.g., timeline follow‐back, hair analyses). As such, it is not clear whether the findings reported here are due to current THC‐bioavailability, long‐term changes, or premorbid vulnerability to CUD. However, a standardized DSM diagnosis can be seen as a more reliable phenotype than (long‐term) frequency of use or cumulative lifetime use (Prince et al., 2018). It is also important to realize that CUD captures those people who not just use, but where use leads to problems, which is potentially a different phenotype regardless of amount of use. Third, results of the resting state analysis could be specific to the parcellation atlas, as low resolution parcellations can lead to unstable estimations. However, individual differences in graph metrics were largely conserved in parcellations with more than 250 regions (Fornito, Zalesky, & Bullmore, 2010), suggesting our findings on a 333‐region cortical parcellation should be stable. Fourth, although our participants were not acutely intoxicated, cognitive functioning and structural/functional connectivity might improve after complete cessation of cannabis use for an extended period of time (Blanco‐Hinojo et al., 2017; D'Souza et al., 2016; Hirvonen et al., 2012; Scott, Slomiak, et al., 2018; Tait et al., 2011). Yet, the required duration of abstinence may depend on the frequency and intensity of use. THC is extremely fat‐soluble and can remain in fatty tissues (including brain) for extensive periods of time (many weeks), especially after chronic use. Last, our sample comprised African Americans, which could limit the generalizability of our findings. However, African Americans are an underrepresented group in psychiatry research while having a higher prevalence of CUD than Caucasians (Wu, Zhu, & Swartz, 2016) and we mostly confirm earlier findings.

In conclusion, in a large sample that was collected irrespective of cannabis use to minimize recruitment bias, we confirm the literature on poorer cognitive functioning in CUD, and an absence of volumetric brain differences between CUD and non‐CUD. We did not find evidence for or against a disruption of structural connectivity. We find disrupted FC in specific regions, although there was sufficient proof that organization of FC as determined via graph metrics does not differ between CUD and non‐user group.

## CONFLICT OF INTERESTS

The authors report no conflicts of interest.

## Supporting information


**Appendix** **S1**. Supporting Information.

## Data Availability

The data that support the findings of this study are available from the corresponding author upon reasonable request.

## References

[hbm25324-bib-0001] Addicott, M. A. , Sweitzer, M. M. , Froeliger, B. , Rose, J. E. , & McClernon, F. J. (2015). Increased functional connectivity in an insula‐based network is associated with improved smoking cessation outcomes. Neuropsychopharmacology, 40(11), 2648–2656. 10.1038/npp.2015.114 25895453 PMC4569957

[hbm25324-bib-0002] Arnone, D. , Barrick, T. R. , Chengappa, S. , Mackay, C. E. , Clark, C. A. , & Abou‐Saleh, M. T. (2008). Corpus callosum damage in heavy marijuana use: Preliminary evidence from diffusion tensor tractography and tract‐based spatial statistics. NeuroImage, 41(3), 1067–1074. 10.1016/j.neuroimage.2008.02.064 18424082

[hbm25324-bib-0003] Ashtari, M. , Cervellione, K. , Cottone, J. , Ardekani, B. A. , & Kumra, S. (2009). Diffusion abnormalities in adolescents and young adults with a history of heavy cannabis use. Journal of Psychiatric Research, 43(3), 189–204. 10.1016/J.JPSYCHIRES.2008.12.002 19111160 PMC3314332

[hbm25324-bib-0004] Becker, M. P. , Collins, P. F. , Lim, K. O. , Muetzel, R. L. , & Luciana, M. (2015). Longitudinal changes in white matter microstructure after heavy cannabis use. Developmental Cognitive Neuroscience, 16, 23–35. 10.1016/j.dcn.2015.10.004 26602958 PMC4691379

[hbm25324-bib-0005] Bidwell, L. C. , Mueller, R. , YorkWilliams, S. L. , Hagerty, S. , Bryan, A. D. , & Hutchison, K. E. (2018). A novel observational method for assessing acute responses to cannabis: Preliminary validation using legal market strains. Cannabis and Cannabinoid Research, 3(1), 35–44. 10.1089/CAN.2017.0038 29607409 PMC5870063

[hbm25324-bib-0006] Blanco‐Hinojo, L. , Pujol, J. , Harrison, B. J. , Macià, D. , Batalla, A. , Nogué, S. , … Martín‐Santos, R. (2017). Attenuated frontal and sensory inputs to the basal ganglia in cannabis users. Addiction Biology, 22(4), 1036–1047. 10.1111/adb.12370 26934839

[hbm25324-bib-0007] Blest‐Hopley, G. , Giampietro, V. , & Bhattacharyya, S. (2018). Residual effects of cannabis use in adolescent and adult brains — A meta‐analysis of fMRI studies. Neuroscience and Biobehavioral Reviews, 88, 26–41. 10.1016/j.neubiorev.2018.03.008 29535069

[hbm25324-bib-0008] Broyd, S. J. , van Hell, H. H. , Beale, C. , Yücel, M. , & Solowij, N. (2016). Acute and chronic effects of cannabinoids on human cognition—A systematic review. Biological Psychiatry, 79(7), 557–567. 10.1016/J.BIOPSYCH.2015.12.002 26858214

[hbm25324-bib-0009] Burns, H. D. , Van Laere, K. , Sanabria‐Bohorquez, S. , Hamill, T. G. , Bormans, G. , Eng, W. ‐s. , … Hargreaves, R. J. (2007). [18F]MK‐9470, a positron emission tomography (PET) tracer for in vivo human PET brain imaging of the cannabinoid‐1 receptor. Proceedings of the National Academy of Sciences, 104(23), 9800–9805. 10.1073/pnas.0703472104 PMC1877985 17535893

[hbm25324-bib-0010] Button, K. S. , Ioannidis, J. P. A. , Mokrysz, C. , Nosek, B. A. , Flint, J. , Robinson, E. S. J. , & Munafò, M. R. (2013). Power failure: Why small sample size undermines the reliability of neuroscience. Nature Reviews Neuroscience, 14(5), 365–376. 10.1038/nrn3475 23571845

[hbm25324-bib-0011] Cheng, H. , Skosnik, P. , Pruce, B. , Brumbaugh, M. , Vollmer, J. , Fridberg, D. , … Newman, S. (2014). Resting state functional magnetic resonance imaging reveals distinct brain activity in heavy cannabis users – A multi‐voxel pattern analysis. Journal of Psychopharmacology, 28(11), 1030–1040. 10.1177/0269881114550354 25237118 PMC4427512

[hbm25324-bib-0012] Chye, Y. , Lorenzetti, V. , Suo, C. , Batalla, A. , Cousijn, J. , Goudriaan, A. E. , … Solowij, N. (2019). Alteration to hippocampal volume and shape confined to cannabis dependence: A multi‐site study. Addiction Biology, 24(4), 822–834. 10.1111/adb.12652 30022573

[hbm25324-bib-0013] Chye, Y. , Suo, C. , Lorenzetti, V. , Batalla, A. , Cousijn, J. , Goudriaan, A. E. , … Yücel, M. (2019). Cortical surface morphology in long‐term cannabis users: A multi‐site MRI study. European Neuropsychopharmacology, 29(2), 257–265. 10.1016/j.euroneuro.2018.11.1110 30558823

[hbm25324-bib-0014] Compton, W. M. , Han, B. , Jones, C. M. , Blanco, C. , & Hughes, A. (2016). Marijuana use and use disorders in adults in the USA, 2002–14: Analysis of annual cross‐sectional surveys. The Lancet Psychiatry, 3(10), 954–964. 10.1016/S2215-0366(16)30208-5 27592339

[hbm25324-bib-0015] Crane, N. A. , Schuster, R. M. , Fusar‐Poli, P. , & Gonzalez, R. (2013). Effects of cannabis on neurocognitive functioning: Recent advances, neurodevelopmental influences, and sex differences. Neuropsychology Review, 23(2), 117–137. 10.1007/s11065-012-9222-1 23129391 PMC3593817

[hbm25324-bib-0016] Defoe, I. N. , Khurana, A. , Betancourt, L. M. , Hurt, H. , & Romer, D. (2018). Disentangling longitudinal relations between youth cannabis use, peer cannabis use, and conduct problems: Developmental cascading links to cannabis use disorder. Addiction (Abingdon, England). 10.1111/add.14456 PMC6519359 30457181

[hbm25324-bib-0017] Delgado‐Rodriguez, M. , & Llorca, J. (2004). Bias. Journal of Epidemiology & Community Health, 58(8), 635–641. 10.1136/jech.2003.008466 15252064 PMC1732856

[hbm25324-bib-0018] Desikan, R. S. , Ségonne, F. , Fischl, B. , Quinn, B. T. , Dickerson, B. C. , Blacker, D. , … Killiany, R. J. (2006). An automated labeling system for subdividing the human cerebral cortex on MRI scans into gyral based regions of interest. NeuroImage, 31(3), 968–980. 10.1016/j.neuroimage.2006.01.021 16530430

[hbm25324-bib-0019] Diedrichsen, J. , Balsters, J. H. , Flavell, J. , Cussans, E. , & Ramnani, N. (2009). A probabilistic MR atlas of the human cerebellum. NeuroImage, 46(1), 39–46. 10.1016/j.neuroimage.2009.01.045 19457380

[hbm25324-bib-0020] D'Souza, D. C. , Cortes‐Briones, J. A. , Ranganathan, M. , Thurnauer, H. , Creatura, G. , Surti, T. , … Skosnik, P. D. (2016). Rapid changes in CB1 receptor availability in cannabis dependent males after abstinence from cannabis. Biological Psychiatry: Cognitive Neuroscience and Neuroimaging, 1(1), 60–67. 10.1016/j.bpsc.2015.09.008 PMC4742341 29560896

[hbm25324-bib-0021] ElSohly, M. A. , Mehmedic, Z. , Foster, S. , Gon, C. , Chandra, S. , & Church, J. C. (2016). Changes in cannabis potency over the last 2 decades (1995–2014): Analysis of current data in the United States. Biological Psychiatry, 79(7), 613–619. 10.1016/j.biopsych.2016.01.004 26903403 PMC4987131

[hbm25324-bib-0022] ElSohly, M. A. , Ross, S. A. , Mehmedic, Z. , Arafat, R. , Yi, B. , & Banahan, B. F. (2000). Potency trends of delta9‐THC and other cannabinoids in confiscated marijuana from 1980–1997. Journal of Forensic Sciences, 45(1), 24–30. Retrieved from. http://www.ncbi.nlm.nih.gov/pubmed/10641915 10641915

[hbm25324-bib-0023] Englund, A. , Atakan, Z. , Kralj, A. , Tunstall, N. , Murray, R. , & Morrison, P. (2016). The effect of five day dosing with THCV on THC‐induced cognitive, psychological and physiological effects in healthy male human volunteers: A placebo‐controlled, double‐blind, crossover pilot trial. Journal of Psychopharmacology, 30(2), 140–151. 10.1177/0269881115615104 26577065

[hbm25324-bib-0024] Epstein, K. A. , & Kumra, S. (2015). White matter fractional anisotropy over two time points in early onset schizophrenia and adolescent cannabis use disorder: A naturalistic diffusion tensor imaging study. Psychiatry Research: Neuroimaging, 232(1), 34–41. 10.1016/j.pscychresns.2014.10.010 25779033

[hbm25324-bib-0025] Esteban, O. , Birman, D. , Schaer, M. , Koyejo, O. O. , Poldrack, R. A. , & Gorgolewski, K. J. (2017). MRIQC: Advancing the automatic prediction of image quality in MRI from unseen sites. PLoS One, 12(9), e0184661. 10.1371/journal.pone.0184661 28945803 PMC5612458

[hbm25324-bib-0026] Filbey, F. M. , Gohel, S. , Prashad, S. , & Biswal, B. B. (2018). Differential associations of combined vs. isolated cannabis and nicotine on brain resting state networks. Brain Structure and Function, 223(7), 3317–3326. 10.1007/s00429-018-1690-5 29882015 PMC6286234

[hbm25324-bib-0027] First, M. B. , Spitzer, R. L. , Gibbon, M. , & Williams, J. B. W. (2002). Structured clinical interview for DSM‐IV‐TR Axis I disorders ‐ patient edition (SCIP‐I/P, 11/2002 revision). New York: Biometrics Research Department, New York State Psychiatric Institute.

[hbm25324-bib-0028] Fischl, B. (2012). FreeSurfer. NeuroImage, 62(2), 774–781. 10.1016/j.neuroimage.2012.01.021 22248573 PMC3685476

[hbm25324-bib-0029] Fornito, A. , Zalesky, A. , & Bullmore, E. T. (2010). Network scaling effects in graph analytic studies of human resting‐state fMRI data. Frontiers in Systems Neuroscience, 4, 22. 10.3389/fnsys.2010.00022 20592949 PMC2893703

[hbm25324-bib-0030] Gillespie, N. A. , Neale, M. C. , Bates, T. C. , Eyler, L. T. , Fennema‐Notestine, C. , Vassileva, J. , … Wright, M. J. (2018). Testing associations between cannabis use and subcortical volumes in two large population‐based samples. Addiction, 113(9), 1661–1672. 10.1111/add.14252 PMC6200645 29691937

[hbm25324-bib-0031] Glass, M. , Faull, R. L. M. L. , & Dragunow, M. (1997). Cannabinoid receptors in the human brain: A detailed anatomical and quantitative autoradiographic study in the fetal, neonatal and adult human brain. Neuroscience, 77(2), 299–318. 9472392 10.1016/s0306-4522(96)00428-9

[hbm25324-bib-0032] Glasser, M. F. , Smith, S. M. , Marcus, D. S. , Andersson, J. L. R. , Auerbach, E. J. , Behrens, T. E. J. , … Van Essen, D. C. (2016). The human connectome Project's neuroimaging approach. Nature Neuroscience, 19(9), 1175–1187. 10.1038/nn.4361 27571196 PMC6172654

[hbm25324-bib-0033] Glasser, M. F. , Sotiropoulos, S. N. , Wilson, J. A. , Coalson, T. S. , Fischl, B. , Andersson, J. L. , … Jenkinson, M. (2013). The minimal preprocessing pipelines for the human connectome project. NeuroImage, 80, 105–124. 10.1016/j.neuroimage.2013.04.127 23668970 PMC3720813

[hbm25324-bib-0034] Gordon, E. M. , Laumann, T. O. , Adeyemo, B. , Huckins, J. F. , Kelley, W. M. , & Petersen, S. E. (2016). Generation and evaluation of a cortical area Parcellation from resting‐state correlations. Cerebral Cortex, 26(1), 288–303. 10.1093/cercor/bhu239 25316338 PMC4677978

[hbm25324-bib-0035] Gruber, S. A. , Dahlgren, M. K. , Sagar, K. A. , Gönenç, A. , & Lukas, S. E. (2014). Worth the wait: Effects of age of onset of marijuana use on white matter and impulsivity. Psychopharmacology, 231(8), 1455–1465. 10.1007/S00213-013-3326-Z 24190588 PMC3967072

[hbm25324-bib-0036] Gruber, S. A. , Sagar, K. A. , Dahlgren, M. K. , Racine, M. , & Lukas, S. E. (2012). Age of onset of marijuana use and executive function. Psychology of Addictive Behaviors: Journal of the Society of Psychologists in Addictive Behaviors, 26(3), 496–506. 10.1037/a0026269 22103843 PMC3345171

[hbm25324-bib-0137] Gur, R. , Ragland, J. D. , Moberg, P. J. , Turner, T. H. , Bilker, W. B. , Kohler, C. , … Gur, R. E. (2001). Computerized neurocognitive scanning: I. methodology and validation in healthy people. Neuropsychopharmacology, 25, 766–776. 10.1016/S0893-133X(01)00278-0 11682260

[hbm25324-bib-0037] Hasin, D. S. , Sarvet, A. L. , Cerdá, M. , Keyes, K. M. , Stohl, M. , Galea, S. , & Wall, M. M. (2017). US adult illicit cannabis use, cannabis use disorder, and medical marijuana Laws: 1991‐1992 to 2012‐2013. JAMA Psychiatry, 74(6), 579–588. 10.1001/jamapsychiatry.2017.0724 28445557 PMC5539836

[hbm25324-bib-0038] Hirvonen, J. , Goodwin, R. S. , Li, C.‐T. , Terry, G. E. , Zoghbi, S. S. , Morse, C. , … Innis, R. B. (2012). Reversible and regionally selective downregulation of brain cannabinoid CB1 receptors in chronic daily cannabis smokers. Molecular Psychiatry, 17(6), 642–649. 10.1038/mp.2011.82 21747398 PMC3223558

[hbm25324-bib-0039] Hua, K. , Zhang, J. , Wakana, S. , Jiang, H. , Li, X. , Reich, D. S. , … Mori, S. (2008). Tract probability maps in stereotaxic spaces: Analyses of white matter anatomy and tract‐specific quantification. NeuroImage, 39(1), 336–347. 10.1016/j.neuroimage.2007.07.053 17931890 PMC2724595

[hbm25324-bib-0040] Jackson, N. J. , Isen, J. D. , Khoddam, R. , Irons, D. , Tuvblad, C. , Iacono, W. G. , … Baker, L. A. (2016). Impact of adolescent marijuana use on intelligence: Results from two longitudinal twin studies. Proceedings of the National Academy of Sciences, 113(5), E500–E508. 10.1073/pnas.1516648113 PMC4747759 26787878

[hbm25324-bib-0237] Jacobus, J. , McQueeny, T. , Bava, S. , Schweinsburg, B. C. , Frank, L. R. , Yang, T. T. , & Tapert, S. F. (2009). White matter integrity in adolescents with histories of marijuana use and binge drinking. Neurotoxicology and Teratology, 31, 349–355. 10.1016/j.ntt.2009.07.006 19631736 PMC2762024

[hbm25324-bib-0041] Jacobus, J. , Goldenberg, D. , Wierenga, C. E. , Tolentino, N. J. , Liu, T. T. , & Tapert, S. F. (2012). Altered cerebral blood flow and neurocognitive correlates in adolescent cannabis users. Psychopharmacology, 222(4), 675–684. 10.1007/s00213-012-2674-4 22395430 PMC3510003

[hbm25324-bib-0042] Jacobus, J. , Squeglia, L. M. , Bava, S. , & Tapert, S. F. (2013). White matter characterization of adolescent binge drinking with and without co‐occurring marijuana use: A 3‐year investigation. Psychiatry Research, 214(3), 374–381. 10.1016/j.pscychresns.2013.07.014 24139957 PMC3900025

[hbm25324-bib-0043] Jakabek, D. , Yücel, M. , Lorenzetti, V. , & Solowij, N. (2016). An MRI study of white matter tract integrity in regular cannabis users: Effects of cannabis use and age. Psychopharmacology, 233(19–20), 3627–3637. 10.1007/s00213-016-4398-3 27503373

[hbm25324-bib-0044] Jarosz, A. F. , & Wiley, J. (2014). What are the odds? A practical guide to computing and reporting Bayes factors. The Journal of Problem Solving, 7(1), 2–9. 10.7771/1932-6246.1167

[hbm25324-bib-0045] Jeffreys, H. (1961). Theory of probability (3rd ed.). Oxford: Oxford University Press.

[hbm25324-bib-0046] Kopec, J. A. , & Esdaile, J. M. (1990). Bias in case‐control studies. A review. Journal of Epidemiology and Community Health, 44(3), 179–186. 10.1136/jech.44.3.179 2273353 PMC1060638

[hbm25324-bib-0047] Liao, Y. , Tang, J. , Liu, T. , Chen, X. , & Hao, W. (2012). Differences between smokers and non‐smokers in regional gray matter volumes: A voxel‐based morphometry study. Addiction Biology, 17(6), 977–980. 10.1111/j.1369-1600.2010.00250.x 20731627

[hbm25324-bib-0048] Lorenzetti, V. , Solowij, N. , & Yücel, M. (2016). The role of cannabinoids in neuroanatomic alterations in cannabis users. Biological Psychiatry, 79(7), e17–e31. 10.1016/J.BIOPSYCH.2015.11.013 26858212

[hbm25324-bib-0049] Lynskey, M. , & Hall, W. (2000). The effects of adolescent cannabis use on educational attainment: A review. Addiction, 95(11), 1621–1630. 10.1046/j.1360-0443.2000.951116213.x 11219366

[hbm25324-bib-0050] Manza, P. , Yuan, K. , Shokri‐Kojori, E. , Tomasi, D. , & Volkow, N. D. (2019). Brain structural changes in cannabis dependence: Association with MAGL. Molecular Psychiatry, 25, 3256–3266. 10.1038/s41380-019-0577-z 31695165 PMC7200265

[hbm25324-bib-0051] Mathias, S. R. , Knowles, E. E. M. , Barrett, J. , Leach, O. , Buccheri, S. , Beetham, T. , … Glahn, D. C. (2017). The processing‐speed impairment in psychosis is more than just accelerated aging. Schizophrenia Bulletin, 43(4), 814–823. 10.1093/schbul/sbw168 28062652 PMC5472152

[hbm25324-bib-0052] Meier, M. H. , Caspi, A. , Ambler, A. , Harrington, H. , Houts, R. , Keefe, R. S. E. , … Moffitt, T. E. (2012). Persistent cannabis users show neuropsychological decline from childhood to midlife. Proceedings of the National Academy of Sciences of the United States of America, 109(40), E2657–E2664. 10.1073/pnas.1206820109 22927402 PMC3479587

[hbm25324-bib-0053] Meier, M. H. , Caspi, A. , Danese, A. , Fisher, H. L. , Houts, R. , Arseneault, L. , & Moffitt, T. E. (2018). Associations between adolescent cannabis use and neuropsychological decline: A longitudinal co‐twin control study. Addiction, 113(2), 257–265. 10.1111/add.13946 28734078 PMC5760333

[hbm25324-bib-0054] Meier, M. H. , Schriber, R. A. , Beardslee, J. , Hanson, J. , & Pardini, D. (2019). Associations between adolescent cannabis use frequency and adult brain structure: A prospective study of boys followed to adulthood. Drug and Alcohol Dependence, 202, 191–199. 10.1016/j.drugalcdep.2019.05.012 31357120

[hbm25324-bib-0055] Morey, R. D. , & Rouder, J. N. (2011). Bayes factor approaches for testing interval null hypotheses. Psychological Methods, 16(4), 406–419. 10.1037/a0024377 21787084

[hbm25324-bib-0056] Morey, R. D. , & Rouder, J. N. (2018). BayesFactor: Computation of Bayes Factors for common designs. Retrieved from https://cran.r-project.org/package=BayesFactor

[hbm25324-bib-0057] Morin, J.‐F. G. , Afzali, M. H. , Bourque, J. , Stewart, S. H. , Séguin, J. R. , O'Leary‐Barrett, M. , & Conrod, P. J. (2019). A population‐based analysis of the relationship between substance use and adolescent cognitive development. The American Journal of Psychiatry, 176(2), 98–106. 10.1176/appi.ajp.2018.18020202 30278790

[hbm25324-bib-0058] Murray, R. M. , Englund, A. , Abi‐Dargham, A. , Lewis, D. A. , Di Forti, M. , Davies, C. , … D'Souza, D. C. (2017). Cannabis‐associated psychosis: Neural substrate and clinical impact. Neuropharmacology, 124, 89–104. 10.1016/j.neuropharm.2017.06.018 28634109

[hbm25324-bib-0059] Nader, D. A. , & Sanchez, Z. M. (2018). Effects of regular cannabis use on neurocognition, brain structure, and function: A systematic review of findings in adults. The American Journal of Drug and Alcohol Abuse, 44(1), 4–18. 10.1080/00952990.2017.1306746 28498718

[hbm25324-bib-0060] Naqvi, N. H. , Gaznick, N. , Tranel, D. , & Bechara, A. (2014). The insula: A critical neural substrate for craving and drug seeking under conflict and risk. Annals of the New York Academy of Sciences, 1316, 53–70. 10.1111/nyas.12415 24690001 PMC4114146

[hbm25324-bib-0061] Orr, C. , Morioka, R. , Behan, B. , Datwani, S. , Doucet, M. , Ivanovic, J. , … Garavan, H. (2013). Altered resting‐state connectivity in adolescent cannabis users. The American Journal of Drug and Alcohol Abuse, 39(6), 372–381. 10.3109/00952990.2013.848213 24200207

[hbm25324-bib-0062] Orr, J. M. , Paschall, C. J. , & Banich, M. T. (2016). Recreational marijuana use impacts white matter integrity and subcortical (but not cortical) morphometry. NeuroImage: Clinical, 12, 47–56. 10.1016/J.NICL.2016.06.006 27408790 PMC4925620

[hbm25324-bib-0063] Owens, M. M. , Sweet, L. H. , & MacKillop, J. (2020). Recent cannabis use is associated with smaller hippocampus volume: High‐resolution segmentation of structural subfields in a large non‐clinical sample. Addiction Biology, 26, e12874. 10.1111/adb.12874 31991525 PMC9187039

[hbm25324-bib-0064] Pearlson, G. D. (2020). Chapter 7 ‐ epidemiology. In Weed science ‐ cannabis controversies and challenges (pp. 135–158). Cambridge, MA: Elsevier/Academic Press.

[hbm25324-bib-0065] Prince, M. A. , Conner, B. T. , & Pearson, M. R. (2018). Quantifying cannabis: A field study of marijuana quantity estimation. Psychology of Addictive Behaviors: Journal of the Society of Psychologists in Addictive Behaviors, 32(4), 426–433. 10.1037/adb0000370 29771542 PMC6013381

[hbm25324-bib-0066] R Core Team . (2017). R: A language and environment for statistical computing. Vienna, Austria: R Foundation for Statistical Computing. Retrieved from. http://www.r-project.org/

[hbm25324-bib-0067] Ranganathan, M. , Radhakrishnan, R. , Addy, P. H. , Schnakenberg‐Martin, A. M. , Williams, A. H. , Carbuto, M. , … D'Souza, D. C. (2017). Tetrahydrocannabinol (THC) impairs encoding but not retrieval of verbal information. Progress in Neuro‐Psychopharmacology and Biological Psychiatry, 79, 176–183. 10.1016/j.pnpbp.2017.06.019 28642081

[hbm25324-bib-0068] Rocchetti, M. , Crescini, A. , Borgwardt, S. , Caverzasi, E. , Politi, P. , Atakan, Z. , & Fusar‐Poli, P. (2013). Is cannabis neurotoxic for the healthy brain? A meta‐analytical review of structural brain alterations in non‐psychotic users. Psychiatry and Clinical Neurosciences, 67(7), 483–492. 10.1111/pcn.12085 24118193

[hbm25324-bib-0069] Ross, J. M. , Ellingson, J. M. , Rhee, S. H. , Hewitt, J. K. , Corley, R. P. , Lessem, J. M. , & Friedman, N. P. (2019). Investigating the causal effect of cannabis use on cognitive function with a quasi‐experimental co‐twin design. Drug and Alcohol Dependence, 10, 7712. 10.1016/J.DRUGALCDEP.2019.107712 PMC7179798 31753729

[hbm25324-bib-0070] Rubinov, M. , & Sporns, O. (2011). Weight‐conserving characterization of complex functional brain networks. NeuroImage, 56(4), 2068–2079. 10.1016/j.neuroimage.2011.03.069 21459148

[hbm25324-bib-0071] Sarvet, A. L. , Wall, M. M. , Fink, D. S. , Greene, E. , Le, A. , Boustead, A. E. , … Hasin, D. S. (2018). Medical marijuana laws and adolescent marijuana use in the United States: A systematic review and meta‐analysis. Addiction, 113(6), 1003–1016. 10.1111/add.14136 29468763 PMC5942879

[hbm25324-bib-0072] Schuster, R. M. , Gilman, J. , Schoenfeld, D. , Evenden, J. , Hareli, M. , Ulysse, C. , … Evins, A. E. (2018). One month of cannabis abstinence in adolescents and young adults is associated with improved memory. The Journal of Clinical Psychiatry, 79(6), 17m11977. 10.4088/JCP.17m11977 PMC6587572 30408351

[hbm25324-bib-0073] Scott, J. C. , Rosen, A. F. G. , Moore, T. M. , Roalf, D. R. , Satterthwaite, T. D. , Calkins, M. E. , … Gur, R. C. (2018). Cannabis Use in Youth is Associated with Limited Alterations in Brain Structure. *BioRxiv*, 443911. 10.1101/443911 PMC6784999 30780151

[hbm25324-bib-0074] Scott, J. C. , Slomiak, S. T. , Jones, J. D. , Rosen, A. F. G. , Moore, T. M. , & Gur, R. C. (2018). Association of cannabis with cognitive functioning in adolescents and young adults. JAMA Psychiatry, 75(6), 585–595. 10.1001/jamapsychiatry.2018.0335 29710074 PMC6137521

[hbm25324-bib-0075] Shollenbarger, S. G. , Price, J. , Wieser, J. , & Lisdahl, K. (2015). Poorer frontolimbic white matter integrity is associated with chronic cannabis use, FAAH genotype, and increased depressive and apathy symptoms in adolescents and young adults. NeuroImage: Clinical, 8, 117–125. 10.1016/j.nicl.2015.03.024 26106535 PMC4473294

[hbm25324-bib-0076] Smith, S. M. , Beckmann, C. F. , Andersson, J. , Auerbach, E. J. , Bijsterbosch, J. , Douaud, G. , … Glasser, M. F. (2013). Resting‐state fMRI in the human connectome project. NeuroImage, 80, 144–168. 10.1016/J.NEUROIMAGE.2013.05.039 23702415 PMC3720828

[hbm25324-bib-0077] Smith, S. M. , Jenkinson, M. , Johansen‐Berg, H. , Rueckert, D. , Nichols, T. E. , Mackay, C. E. , … Behrens, T. E. J. (2006). Tract‐based spatial statistics: Voxelwise analysis of multi‐subject diffusion data. NeuroImage, 31(4), 1487–1505. 10.1016/j.neuroimage.2006.02.024 16624579

[hbm25324-bib-0078] Smith, S. M. , Jenkinson, M. , Woolrich, M. W. , Beckmann, C. F. , Behrens, T. E. J. , Johansen‐Berg, H. , … Matthews, P. M. (2004). Advances in functional and structural MR image analysis and implementation as FSL. NeuroImage, 23(Suppl 1), S208–S219. 10.1016/j.neuroimage.2004.07.051 15501092

[hbm25324-bib-0079] Sotiropoulos, S. N. , Jbabdi, S. , Xu, J. , Andersson, J. L. , Moeller, S. , Auerbach, E. J. , … Behrens, T. E. J. (2013). Advances in diffusion MRI acquisition and processing in the human connectome project. NeuroImage, 80, 125–143. 10.1016/J.NEUROIMAGE.2013.05.057 23702418 PMC3720790

[hbm25324-bib-0080] Stephens, M. , & Balding, D. J. (2009). Bayesian statistical methods for genetic association studies. Nature Reviews Genetics, 10(10), 681–690. 10.1038/nrg2615 19763151

[hbm25324-bib-0081] Tait, R. J. , Mackinnon, A. , & Christensen, H. (2011). Cannabis use and cognitive function: 8‐year trajectory in a young adult cohort. Addiction (Abingdon, England), 106(12), 2195–2203. 10.1111/j.1360-0443.2011.03574.x 21749524

[hbm25324-bib-0082] Thayer, R. E. , YorkWilliams, S. , Karoly, H. C. , Sabbineni, A. , Ewing, S. F. , Bryan, A. D. , & Hutchison, K. E. (2017). Structural neuroimaging correlates of alcohol and cannabis use in adolescents and adults. Addiction, 112(12), 2144–2154. 10.1111/add.13923 28646566 PMC5673530

[hbm25324-bib-0083] Thijssen, S. , Rashid, B. , Gopal, S. , Nyalakanti, P. , Calhoun, V. D. , & Kiehl, K. A. (2017). Regular cannabis and alcohol use is associated with resting‐state time course power spectra in incarcerated adolescents. Drug and Alcohol Dependence, 178, 492–500. 10.1016/J.DRUGALCDEP.2017.05.045 28715777 PMC5561725

[hbm25324-bib-0084] Volkow, N. D. , Baler, R. D. , Compton, W. M. , & Weiss, S. R. B. (2014). Adverse health effects of marijuana use. New England Journal of Medicine, 370(23), 2219–2227. 10.1056/NEJMra1402309 PMC4827335 24897085

[hbm25324-bib-0085] Wang, G. S. , Davies, S. D. , Halmo, L. S. , Sass, A. , & Mistry, R. D. (2018). Impact of marijuana legalization in Colorado on adolescent emergency and urgent care visits. Journal of Adolescent Health, 63(2), 239–241. 10.1016/j.jadohealth.2017.12.010 29609916

[hbm25324-bib-0086] Weiland, B. J. , Thayer, R. E. , Depue, B. E. , Sabbineni, A. , Bryan, A. D. , & Hutchison, K. E. (2015). Daily marijuana use is not associated with brain morphometric measures in adolescents or adults. Journal of Neuroscience, 35(4), 1505–1512. 10.1523/JNEUROSCI.2946-14.2015 25632127 PMC4308597

[hbm25324-bib-0087] Wen, H. , Hockenberry, J. M. , & Druss, B. G. (2019). The effect of medical marijuana Laws on marijuana‐related attitude and perception among US adolescents and young adults. Prevention Science, 20(2), 215–223. 10.1007/s11121-018-0903-8 29767282

[hbm25324-bib-0088] Wu, L.‐T. , Zhu, H. , & Swartz, M. S. (2016). Trends in cannabis use disorders among racial/ethnic population groups in the United States. Drug and Alcohol Dependence, 165, 181–190. 10.1016/j.drugalcdep.2016.06.002 27317045 PMC4939114

[hbm25324-bib-0089] Zalesky, A. , Fornito, A. , & Bullmore, E. T. (2010). Network‐based statistic: Identifying differences in brain networks. NeuroImage, 53(4), 1197–1207. 10.1016/j.neuroimage.2010.06.041 20600983

[hbm25324-bib-0337] Zalesky, A. , Solowij, N. , Yücel, M. , Lubman, D. I. , Takagi, M. , Harding, I. H. , … Seal, M. (2012). Effect of long‐term cannabis use on axonal fibre connectivity. Brain, 135, 2245–2255. 10.1093/brain/aws136 22669080

