# Supplementary material for: Associations of cannabis use disorder with cognition, brain structure, and brain function in African Americans
Source: Hum Brain Mapp. 2020 Dec 19;42(6):1727–41. doi: 10.1002/hbm.25324 (PMC7978126; doi:10.1002/hbm.25324)
Supplement: Supplementary file 1 — Appendix S1. Supporting Information. [file HBM-42-1727-s001.pdf]

SUPPORTING INFORMATION

to

Koenis et al., Associations of cannabis use disorder with cognition, brain structure, and brain function in African Americans

Table of Contents

Supplemental Material and Methods
- Participants 2
- Cognition 2
- Preprocessing of resting state data 2
- Network-based statistic 3
- Graph theory 3

Bayes Factors
- Null interval 4
- Posterior probability of association 4

Supplemental Results
- Correlation with duration of cannabis use and age of first age 4
- Correlation between cognition and brain outcomes 5
- FC results on non-GSR data 5

References 5

Tables
- Table S1
  - S1a. Subjects included in neurocognitive analyses 7
  - S1b. Subjects included in morphometry analyses 8
  - S1c. Subjects included in diffusion imaging analyses 9
  - S1d. Subjects included in resting state analyses 10
- Table S2. Regional cortical thickness (mean (SD)) 11
- Table S3. Regional cortical surface area and subcortical volumes (mean (SD)) 12
- Table S4. Mean (SD) FA of the skeleton and JHU white matter tracts 14
- Table S5. Marginally significant results in regional graph theory metrics (at $p < 0.005$) 15
- Table S6. Marginally significant results in regional graph theory metrics of non-GSR (at $p < 0.005$) 16
- Table S7. Results GSR graph metrics with same density as non-GSR results 17

Figures
- Figure S1. Overview of the number of participants per modality and reasons for exclusion 18
- Figure S2. Correlations between all cognitive measures. 19
- Figure S3. Output of all NBS networks over thresholds F≥ 12 to F≥ 16 20
- Figure S4. Visualisation of the FDR significant edges 21
- Figure S5. The relation between the prior probability, the Bayes factor (BF) and the posterior probability 22

Supplemental Material and Methods

Participants

DSM-IV diagnoses were determined through structured clinical interviews and a consensus process. The process was as follows: research assistants at (minimally) BA level who were trained in diagnostic interviews conducted the SCID and wrote a report. At weekly case conferences, diagnostic experts read the report and the group as a whole agreed upon the diagnosis.

We did not screen for alcohol or psychedelics like LSD and psilocybin. However, only one participant had a diagnosis of substance use disorder for a drug for which we didn't screen (LSD, full sustained remission) and visibly intoxicated participants or participants who mentioned they had a drink before coming in for the research study (as part of the health questionnaire) were excluded. It is therefore unlikely that our results are impacted by participants who had traces of drugs (other than cannabis) in their system.

Years of education were approximated as an ordinal variable as follows: 1) grade 6 or less; 2) grade 7-12 without graduation; 3) high school/GED graduate; 4) part college; 5) 2-yr college/trade school graduate; 6) 4-yr college graduate; 7) part graduate school or professional school; 8) complete graduate or professional school.

Cognition

General intellectual functioning *g* was defined as the first principle component from a PCA analyses done in R with prcomp. To avoid missing data for *g* (because *g* cannot be estimated for participants with missing data on one or more cognitive tests), missing data on any cognitive test was imputed with the median of available data before the computation of *g* with a PCA. The estimate of *g* did not change when using available data or imputed data: % of variance explained (38%) and correlation with WASI IQ (r= 0.64) were the same when imputed missing data or using available data. After PCA, imputed data was reset to NA. Subjects who scored <70 for WASI IQ (but >70 for WTAR IQ) were excluded from cognitive analyses (n= 5 non-CUD, 1 CUD). Eight subjects did not have cognitive data. Final group sizes were 142 non-CUD, 53 CUD (see also Figure S1).

Post hoc, a multiple regression was done to account for the interdependency of the cognitive tasks. This was done in R with glm, with a binomial family, including all variables but *g* (because *g* is computed based on all variables and is thus highly colinear with all variables): glm(cannabis state ~ var1 + var2 + var3 +…+ var 20, family=binomial).

Preprocessing of resting state data

Resting state fMRI data were preprocessed using a pipeline developed at Washington University (Glasser et al., 2013). Preprocessing included distortion removal, motion estimation, cross-modal registration and alignment to standard space. Consequently, for all sessions, the first 10 timepoints were discarded to allow for magnetization stabilization. The data were despiked using AFNI's 3dDespike (Jo et al., 2013). Motion was regressed from the signal using the movement regressors, including location estimates and their derivatives DVARS (Power et al., 2012) and framewise displacement (FD). The motion regression was done using FSL (Smith et al., 2004), and also included variance normalization and demeaning. Global signal regression (GSR, based on all voxels) was applied to remove remaining nuisance signals. In the remaining signal, compcor (Behzadi et al., 2007) was applied to identify high variance voxels and regress their

average signal from the signal using nilearn (Abraham et al., 2014). Bandpass filtering was applied (0.01 - 0.125 Hz). Lastly, frame censoring was applied to frames with FD > 0.5mm, as well as to uncensored segments of data with less than 5 contiguous volumes.

After the data cleaning procedure, average time series were extracted for 382 predefined regions of interest using the Harvard Oxford subcortical atlas for subcortical regions (Desikan et al., 2006), the MNIFnirt atlas for the cerebellum (Diedrichsen et al., 2009), and the Gordon atlas for the cortex (Gordon et al., 2016). From the time series, a parcel by parcel correlation matrix was generated using Pearson's correlation. Lastly, we applied a Fisher's r to Z transformation.

Network Based Statistic

The network-based statistic (Zalesky et al., 2010) procedure includes the following steps:

1) Identify edges that show a group difference in connectivity strength. Group differences in connectivity strength were determined over each edge with a two-sided F-test. We first tested over a large range of thresholds to get a sense of the sensitivity to the thresholds: from $F \geq 4$ (which corresponds to $p < 0.047$, uncorrected) to $F \geq 17$ ($p < 0.000063$), in steps of 1. Then, for interpretability we included ranges that resulted in the largest component having more than 2 but less than 75 edges (regardless of significance of the components), resulting in F≥ 12-16. These thresholds signify strong edge-differences ($p < 0.0007$) between the groups while having meaningful component sizes.
2) From the resulting adjacency matrix, identify all distinct components (subgraphs with at least one path between every node);
3) Rank components in terms of their size, either based on i) number of edges (extent) or ii) sum of F-statistics of all edges (intensity);
4) The size of each component is the test statistic, with a null-distribution generated from the size of the largest component from each of 5000 permutations of group membership. Note that this test has a family wise error correction for multiple comparisons. We also tested for significant differences in the adjacency matrix with FDR correction in the NBS toolbox (50000 permutations, $q < 0.05$).

Graph theory

The following graph-theoretical metrics were estimated:

1) metrics of global connectivity: global efficiency, a measurement of network integration; and clustering coefficient, a measurement of global segregation
2) metrics of regional connectivity: degree, the number of edges connected to the node; strength, the sum of weights of edges connected to the node; and participation coefficient, the extent to which a given node connects to nodes in modules other than its own
3) metrics of modular organization: modularity, the proportion of intra-modular edges over all modules in the network; and community structure, the assignment of regional nodes to specific modules. Differences in community structure were tested with normalized mutual information (partition-distance in BCT).

Bayes Factors

Null interval

A standard null hypothesis used in many statistical tests is that the difference between groups is zero. However, even in the absence of a true effect, the difference between two groups will never be exactly zero. Therefore, it is more appropriate to test against a null-interval (Morey and Rouder, 2011). The null-interval includes non-zero effects that are too small to be of interest. To be able to pick up small deviations from the null, we choose a null-interval of (-0.1, 0.1), where 0.1 is the standardized difference between the groups (i.e. effect size).

Posterior probability

Multiple testing correction was done as discussed by Stephens & Balding (Stephens and Balding, 2009). The basis of the method is the *a priori* estimate (i.e., the prior probability π) of the proportion of tests that are truly associated with CUD. With the prior we can compute the posterior odds as PO = BF * (π / 1 - π) and the posterior probability of association as PPA = PO / (1+PO) (Stephens and Balding, 2009). The PPA can be interpreted directly as a probability of a true effect, irrespective of power, sample size or how many tests were done (Stephens and Balding, 2009). See Figure S5 for a visualization of the relation between BF, prior π and the PPA. From these formulas, we can infer that with a default prior π = 0.5, BF = 3 gives PPA = 0.75, and BF = 0.33 gives PPA = 0.25. Because BF > 3 is considered substantial, and BF > 10 is strong evidence for the alternative (Jeffreys, 1961), we use PPA > 0.75 as substantial evidence, and PPA > 0.90 for strong evidence for a true effect. PPA < 0.25 and 0.10 are substantial and strong evidence for the null hypothesis respectively.

For the graph metrics, this results in the following. Because we use the BF to provide additional information on the model evidence and not as a first method of choice, we assumed the probability to be the number of regions found with the $p < 0.005$ threshold. With π = 4 / (8*382) = 0.0013, PPA for all graph metrics is < 0.01, which is strong evidence for a null finding. When we increase π to 0.01, PPA remains low (PPA < 0.14). Substantial evidence for a real result (PPA > 0.75) would require BF > 2300 with π = 0.0013, or BF = 15 with π = 0.165. A π = 0.165 represents an unrealistic expectation to find more than 63 significant regions per graph metric.

Similarly, for the edge-wise significance only the BF for the insula-SPG gives a high PPA of 0.99 when we assume a (high) prior of 0.001 (that is, 73 significant edges out of the (381*382)/2=72771 edges). The prior of 0.0001 based on the data gives PPA = 0.95. Evidence of an association between CUD and functional connectivity (FC) for the other edges ranges from inconclusive (PPA < 0.42 with a high prior) to strong evidence for the null (PPA < 0.07 with the low data-driven prior). Note that we did not compute BF for NBS-networks as a whole.

We conclude that there is strong evidence for the *absence* of an association between CUD and resting state graph metrics. There is strong evidence for lower FC between the right insula and right superior parietal gyrus in CUD group**.**

Supplemental Results

Correlation with duration of cannabis use and age of first use

None of the cognitive tests correlated with duration of use (all $r < 0.14$, $p > 0.33$, uncorrected) or age of first use ($r < 0.19$, $p > 0.19$). FC of the NBS and FDR edges did not correlate with duration of use ($r < |0.31|$, $p > 0.05$) or age of onset ($r < |0.24|$, $p > 0.14$). BF for all correlations was inconclusive (0.2 > BF < 1.8).

Correlation between significant cognition and brain variables

Correlation between cognition and FC of significant edges was found at $p < 0.005$ for CVLT sum correct with two edges: right SPG to right insula (r= 0.28, p= 0.002, BF= 8.5), and left accumbens to right hippocampus (r = -0.28, p= 0.001, BF= 9.8). The correlations are in the opposite direction of the association of CUD with FC. None of the findings survived FDR correction. Although BF> 3 suggests substantial evidence for a true correlation, the PPA is only 0.15 for BF= 9.8 when considering the multiple tests and a prior probability of 2 / (7*16) = 0.018. Thus, these findings are inconclusive at best.

FC results on non-GSR data

None of the correlations between resting state modules reached FDR significance. Two out of 120 correlations were significant at $p < 0.005$: mean between-network-FC of positive edges was stronger in the CUD group between the cingulo-parietal and somatomotor area of the mouth (p=0.0024, Cohen's d=0.60, BF=13.4), and between the cingulo-parietal and auditory resting state network (p=0.0042, Cohen's d=0.56, BF=7.5). Using this as a prior gives π = 0.017, with PPA=0.19 and 0.11, thus, support for the absence of a group difference. A more lenient prior of π =0.125 results in inconclusive PPA = 0.66 and 0.52. Mean within and outward strength for each of the 15 resting state modules was inconclusive with a lenient π = 0.33, BF < 3.74, PPA <0.65.

For the graph metrics, we used a data driven prior based on the number of results that were significant at $p < 0.005$: with π = 28 / (8*382) = 0.0092, 24 of the variables have PPA < 0.20, suggesting evidence for the absence of an effect. Four variables had a BF >40, with an inconclusive PPA<0.33, see Table S6. For the GSR-results with the same density as the non-GSR (Table S7), π = 13 / (8*382) = 0.0043, resulting in PPA < 0.08, suggesting strong evidence for the absence of an effect.

References

Abraham, A., Pedregosa, F., Eickenberg, M., Gervais, P., Mueller, A., Kossaifi, J., Gramfort, A., Thirion, B., Varoquaux, G., 2014. Machine learning for neuroimaging with scikit-learn. Front. Neuroinform. 8, 14. https://doi.org/10.3389/fninf.2014.00014

Behzadi, Y., Restom, K., Liau, J., Liu, T.T., 2007. A component based noise correction method (CompCor) for BOLD and perfusion based fMRI. Neuroimage 37, 90–101. https://doi.org/10.1016/j.neuroimage.2007.04.042

Desikan, R.S., Ségonne, F., Fischl, B., Quinn, B.T., Dickerson, B.C., Blacker, D., Buckner, R.L., Dale, A.M., Maguire, R.P., Hyman, B.T., Albert, M.S., Killiany, R.J., 2006. An automated labeling system for subdividing the human cerebral cortex on MRI scans into gyral based regions of interest. Neuroimage 31, 968–80. https://doi.org/10.1016/j.neuroimage.2006.01.021

Diedrichsen, J., Balsters, J.H., Flavell, J., Cussans, E., Ramnani, N., 2009. A probabilistic MR atlas of the human cerebellum. Neuroimage 46, 39–46. https://doi.org/10.1016/j.neuroimage.2009.01.045

Glasser, M.F., Sotiropoulos, S.N., Wilson, J.A., Coalson, T.S., Fischl, B., Andersson, J.L., Xu, J., Jbabdi, S., Webster, M., Polimeni, J.R., Van Essen, D.C., Jenkinson, M., 2013. The minimal preprocessing pipelines for the Human Connectome Project. Neuroimage 80, 105–124. https://doi.org/10.1016/j.neuroimage.2013.04.127

Gordon, E.M., Laumann, T.O., Adeyemo, B., Huckins, J.F., Kelley, W.M., Petersen, S.E., 2016. Generation and Evaluation of a Cortical Area Parcellation from Resting-State Correlations. Cereb. Cortex 26, 288–303. https://doi.org/10.1093/cercor/bhu239

Jeffreys, H., 1961. Theory of Probability, 3rd ed. Oxford University Press, Oxford.

Jo, H.J., Gotts, S.J., Reynolds, R.C., Bandettini, P.A., Martin, A., Cox, R.W., Saad, Z.S., 2013. Effective Preprocessing Procedures Virtually Eliminate Distance-Dependent Motion Artifacts in Resting State FMRI. J. Appl. Math. 2013. https://doi.org/10.1155/2013/935154

Morey, R.D., Rouder, J.N., 2011. Bayes factor approaches for testing interval null hypotheses. Psychol. Methods 16,

406–19. https://doi.org/10.1037/a0024377
Power, J.D., Barnes, K.A., Snyder, A.Z., Schlaggar, B.L., Petersen, S.E., 2012. Spurious but systematic correlations in functional connectivity MRI networks arise from subject motion. Neuroimage 59, 2142–2154. https://doi.org/10.1016/J.NEUROIMAGE.2011.10.018
Smith, S.M., Jenkinson, M., Woolrich, M.W., Beckmann, C.F., Behrens, T.E.J., Johansen-Berg, H., Bannister, P.R., De Luca, M., Drobnjak, I., Flitney, D.E., Niazy, R.K., Saunders, J., Vickers, J., Zhang, Y., De Stefano, N., Brady, J.M., Matthews, P.M., 2004. Advances in functional and structural MR image analysis and implementation as FSL. Neuroimage 23 Suppl 1, S208-19. https://doi.org/10.1016/j.neuroimage.2004.07.051
Stephens, M., Balding, D.J., 2009. Bayesian statistical methods for genetic association studies. Nat. Rev. Genet. 10, 681–690. https://doi.org/10.1038/nrg2615
Xia, M., Wang, J., He, Y., 2013. BrainNet Viewer: a network visualization tool for human brain connectomics. PLoS One 8, e68910. https://doi.org/10.1371/journal.pone.0068910
Zalesky, A., Fornito, A., Bullmore, E.T., 2010. Network-based statistic: Identifying differences in brain networks. Neuroimage 53, 1197–1207. https://doi.org/10.1016/j.neuroimage.2010.06.041

Table S1a. Subjects included in neurocognitive analyses

	non-CUD	CUD	*p*
N	142	53	
Male, n (%)	60 (42%)	28 (53%)	0.199 [3]
Age (range)	40 (18-70)	37 (19-69)	0.198 [4]
Duration of CB use, mean (range in yrs)[1]	-	11 (1-40)	
Education, median (range)	4 (2-8)	3 (2-6)	<0.001[5]
On social disability, n (%)[1]	16 (11.3%)	2 (3.8%)	0.163[3]
Axis I Psychiatric Diagnoses, n (%)			
No diagnosis	92 (64.79%)	-	
Depression	13 (9.15%)	8 (15.09%)	0.298 [3]
PTSD	12 (8.45%)	3 (5.66%)	0.763 [3]
Anxiety disorders	5 (3.52%)	4 (7.55%)	0.258 [3]
ADHD	0 (0%)	1 (1.89%)	0.272 [3]
Alcohol abuse/dependence	28 (19.72%)	20 (37.74%)	0.014 [3]
Drug other than CB abuse/dependence	14 (9.86%)	19 (35.85%)	0.026 [3]
Medication, n (%)[2]			
Anxiolytic	3 (2.07%)	0 (0%)	0.564 [3]
Antidepressant	5 (3.45%)	2 (3.77%)	1 [3]
Atypical antipsychotic [2]	2 (1.38%)	0 (0%)	1 [3]
Anticonvulsant [2]	2 (1.38%)	0 (0%)	1 [3]
Alcohol & Nicotine			
Nr drinks per month, mean (sd)	9 (19)	13 (16)	0.001 [4]
Currently smoking, n (%)	43 (30.28)	38 (71.70)	< 0.001 [3]
FTND, median (range) [1]	0 (0-9)	3 (0-9)	< 0.001 [5]

Diagnoses are lifetime diagnoses. CB= cannabis; FTND= Fagerström test for nicotine dependence (ranges from 0 to 12).

[1] missing data for cannabis duration (n=2); social security (n=6 non-CUD; 2 CUD); FTND (n=36, equal percentage in the non-CUD & CUD group)

[2] None of the participants used mood stabilizers or lithium. Atypical antipsychotic medication was used for treatment of MDD; Anticonvulsant was used for nerve pain.

[3] Fisher's exact test

[4] Welch's *t* test

[5] Wilcoxon rank sum test

Table S1b. Subjects included in neuroanatomy analyses.

	non-CUD	CUD	*p*
N	107	45	
Male, n (%)	51 (48%)	22 (49%)	1.000 [3]
Age (range)	40 (19-70)	37 (19-69)	0.280 [4]
Duration of CB use, mean (range in yrs)[1]	-	10 (1-35)	
Education, median (range)	4 (2-8)	3 (2-6)	0.002[5]
On social disability, n (%)	11 (10.3%)	2 (4.4%)	0.347[3]
Axis I Psychiatric Diagnoses, n (%)			
No diagnosis	66 (61.68%)	-	
Depression	10 (9.35%)	7 (15.56%)	0.395 [3]
PTSD	9 (8.41%)	2 (4.44%)	0.610 [3]
Anxiety disorders	5 (4.67%)	3 (6.67%)	0.758 [3]
ADHD	0 (0%)	1 (2.22%)	0.395 [3]
Alcohol abuse/dependence	23 (21.50%)	16 (35.56%)	0.246 [3]
Drug other than CB abuse/dependence	11 (10.28%)	16 (35.56%)	0.280 [3]
Medication, n (%)[2]			
Anxiolytic	2 (1.83%)	0 (0)	1 [3]
Antidepressant	3 (2.75%)	2 (4.44%)	0.633 [3]
Atypical antipsychotic [2]	2 (1.83%)	0 (0)	1 [3]
Anticonvulsant [2]	2 (1.83%)	0 (0)	1 [3]
Alcohol & Nicotine			
Nr drinks per month, mean (sd)	8 (18)	13 (16)	0.002 [4]
Currently smoking, n (%)	31 (28.97)	32 (71.11)	< 0.001 [3]
FTND, median (range) [1]	0 (0-9)	3 (0-9)	0.009 [5]

Diagnoses are lifetime diagnoses. CB= cannabis; FTND= Fagerström test for nicotine dependence (ranges from 0 to 12).
Bold: significant difference ($p < 0.05$)
[1] missing data for cannabis duration (1); FTND (24, equal percentage in the non-CUD & CUD group),
[2] None of the participants used mood stabilizers or lithium. Atypical antipsychotic medication was used for treatment of MDD; Anticonvulsant was used for nerve pain.
[3] Fisher's exact test
[4] Welch's *t* test
[5] Wilcoxon rank sum test

Table S1c. Subjects included in diffusion imaging analyses.

	non-CUD	CUD	*p*
N	110	42	
Male, n (%)	50 (45%)	21 (50%)	0.547 [3]
Age (range)	40 (19-69)	38 (19-69)	0.717 [4]
Duration of CB use, mean (range in yrs)	-	11 (1-35)	
Education, median (range)	4 (2-8)	3 (2-6)	<0.001[5]
On social disability, n (%)	9 (8.2%)	2 (4.8%)	0.728[3]
Axis I Psychiatric Diagnoses, n (%)			
No diagnosis	70 (63.64%)	-	
Depression	9 (8.18%)	7 (16.67%)	0.145 [3]
PTSD	9 (8.18%)	2 (4.76%)	0.728 [3]
Anxiety disorders	5 (4.55%)	4 (9.52%)	0.262 [3]
ADHD	0 (0%)	1 (2.38%)	0.276 [3]
Alcohol abuse/dependence	26 (23.64%)	14 (33.33%)	0.225 [3]
Drug other than CB abuse/dependence	10 (9.09%)	16 (38.1%)	0.146 [3]
Medication, n (%)[1]			
Anxiolytic	1 (0.90%)	0 (0%)	1 [3]
Antidepressant	3 (2.70%)	2 (4.76%)	0.617 [3]
Atypical antipsychotic [1]	2 (1.80%)	0 (0%)	1 [3]
Anticonvulsant [1]	2 (1.80%)	0 (0%)	1 [3]
Alcohol & Nicotine			
Nr drinks per month, mean (sd)	8 (18)	14 (17)	0.002 [4]
Currently smoking, n (%)	30 (27.27)	29 (69.05)	< 0.001 [3]
FTND, median (range) [2]	0 (0-9)	3 (0-9)	0.008 [5]

Diagnoses are lifetime diagnoses. CB= cannabis; FTND= Fagerström test for nicotine dependence (ranges from 0 to 12).
Bold: significant difference ($p < 0.05$)
[1] None of the participants used mood stabilizers or lithium. Atypical antipsychotic medication was used for treatment of MDD; Anticonvulsant was used for nerve pain.
[2] missing data for FTND (n=24, equal percentage in the non-CUD & CUD group)
[3] Fisher's exact test
[4] Welch's *t* test
[5] Wilcoxon rank sum test

Table S1d. Subjects included in resting state analyses.

	non-CUD	CUD	*p*
N	92	39	
Male, n (%)	43 (47%)	19 (49%)	0.851 [3]
Age (range)	39 (19-70)	34 (19-60)	0.096 [4]
Duration of CB use, mean (range in yrs)	-	11 (1-35)	
Education, median (range)	4 (2-8)	3 (2-6)	<0.001[5]
On social disability, n (%)	8 (8.7%)	1 (2.6%)	0.279 [3]
Axis I Psychiatric Diagnoses, n (%)			
No diagnosis	56 (60.87%)	-	
Depression	9 (9.78%)	4 (10.26%)	1.000 [3]
PTSD	9 (9.78%)	2 (5.13%)	0.505 [3]
Anxiety disorders	5 (5.43%)	3 (7.69%)	0.695 [3]
ADHD	0 (0%)	1 (2.56%)	0.298 [3]
Alcohol abuse/dependence	21 (22.83%)	12 (30.77%)	0.381 [3]
Drug other than CB abuse/dependence	8 (8.70%)	14 (35.9%)	0.436 [3]
Medication, n (%) [1]			
Anxiolytic	2 (2.13%)	0 (0%)	1 [3]
Antidepressant	2 (2.13%)	2 (5.13%)	0.285 [3]
Atypical antipsychotic [1]	2 (2.13%)	0 (0%)	1 [3]
Anticonvulsant [1]	1 (1.06%)	0 (0%)	1 [3]
Alcohol & Nicotine			
Nr drinks per month, mean (sd)	9 (19)	11 (15)	0.006 [4]
Currently smoking, n (%)	26 (28.26)	28 (71.79)	< 0.001 [3]
FTND, median (range) [2]	0 (0-9)	3 (0-9)	0.002 [5]

Diagnoses are lifetime diagnoses. CB= cannabis; FTND= Fagerström test for nicotine dependence (ranges from 0 to 12).
Bold: significant difference ($p < 0.05$)
[1] None of the participants used mood stabilizers or lithium. Atypical antipsychotic medication was used for treatment of MDD; Anticonvulsant was used for nerve pain.
[2] missing data for FTND (24, equal percentage in the non-CUD & CUD group)
[3] Fisher’s exact test
[4] Welch’s *t* test
[5] Wilcoxon rank sum test

Table S2. Regional cortical thickness (mean (SD)).

		Left					Right				
lobe	Region (thickness)	non-CUD	CUD	Cohen's *d*	*p*	BF	non-CUD	CUD	Cohen's *d*	*p*	BF
frontal	caudalmiddlefrontal	2.67 (0.20)	2.70 (0.23)	-0.06	0.76	0.13	2.69 (0.19)	2.67 (0.20)	-0.35	0.06	0.91
	frontalpole	2.81 (0.31)	2.94 (0.36)	0.33	0.09	0.80	2.83 (0.31)	2.83 (0.32)	-0.16	0.41	0.21
	lateralorbitofrontal	2.71 (0.22)	2.73 (0.17)	-0.08	0.62	0.14	2.70 (0.24)	2.75 (0.19)	0.10	0.55	0.15
	medialorbitofrontal	2.55 (0.22)	2.54 (0.23)	-0.18	0.35	0.23	2.72 (0.21)	2.72 (0.20)	-0.13	0.50	0.17
	paracentral	2.46 (0.16)	2.48 (0.16)	-0.05	0.79	0.13	2.48 (0.18)	2.51 (0.19)	0.05	0.79	0.13
	parsopercularis	2.72 (0.23)	2.75 (0.21)	-0.09	0.61	0.14	2.77 (0.21)	2.83 (0.18)	0.17	0.32	0.22
	parsorbitalis	2.74 (0.30)	2.81 (0.23)	0.15	0.37	0.19	2.78 (0.29)	2.85 (0.22)	0.15	0.37	0.19
	parstriangularis	2.57 (0.21)	2.63 (0.21)	0.16	0.39	0.21	2.63 (0.23)	2.69 (0.19)	0.12	0.47	0.17
	precentral	2.63 (0.21)	2.64 (0.21)	-0.08	0.64	0.14	2.62 (0.22)	2.63 (0.24)	-0.12	0.53	0.16
	rostralmiddlefrontal	2.52 (0.19)	2.54 (0.19)	-0.12	0.51	0.16	2.54 (0.19)	2.58 (0.18)	0.08	0.66	0.14
	superiorfrontal	2.80 (0.20)	2.82 (0.24)	-0.06	0.76	0.13	2.81 (0.21)	2.83 (0.21)	-0.09	0.62	0.15
medial temp	entorhinal	3.01 (0.35)	3.01 (0.30)	-0.07	0.70	0.13	3.13 (0.34)	3.17 (0.35)	0.07	0.72	0.13
	fusiform	2.75 (0.16)	2.77 (0.14)	-0.01	0.93	0.12	2.77 (0.16)	2.80 (0.14)	0.04	0.81	0.13
	parahippocampal	2.56 (0.30)	2.57 (0.26)	-0.08	0.67	0.14	2.53 (0.28)	2.57 (0.23)	0.08	0.67	0.14
	temporalpole	3.20 (0.44)	3.23 (0.35)	0.02	0.90	0.12	3.32 (0.43)	3.38 (0.36)	0.10	0.54	0.15
lateral temp	bankssts	2.59 (0.18)	2.64 (0.15)	0.15	0.37	0.19	2.71 (0.21)	2.79 (0.18)	0.31	0.07	0.66
	inferiortemporal	2.85 (0.20)	2.86 (0.17)	-0.09	0.61	0.14	2.88 (0.21)	2.89 (0.15)	-0.02	0.89	0.12
	middletemporal	2.89 (0.23)	2.93 (0.18)	0.05	0.75	0.13	2.96 (0.21)	3.01 (0.16)	0.16	0.31	0.21
	superiortemporal	2.79 (0.23)	2.83 (0.18)	0.05	0.75	0.13	2.85 (0.22)	2.89 (0.17)	0.05	0.76	0.13
	transversetemporal	2.53 (0.22)	2.53 (0.23)	-0.10	0.58	0.15	2.56 (0.28)	2.55 (0.20)	-0.19	0.24	0.25
parietal	inferiorparietal	2.54 (0.15)	2.57 (0.15)	0.11	0.51	0.16	2.56 (0.17)	2.59 (0.17)	0.04	0.83	0.13
	postcentral	2.13 (0.14)	2.14 (0.12)	0.00	0.98	0.12	2.16 (0.15)	2.17 (0.14)	-0.08	0.66	0.14
	precuneus	2.46 (0.15)	2.48 (0.14)	0.01	0.96	0.12	2.48 (0.14)	2.53 (0.14)	0.28	0.14	0.48
	superiorparietal	2.24 (0.11)	2.27 (0.13)	0.10	0.59	0.15	2.25 (0.14)	2.28 (0.14)	0.10	0.58	0.15
	supramarginal	2.59 (0.18)	2.62 (0.16)	0.03	0.88	0.12	2.61 (0.18)	2.65 (0.15)	0.08	0.65	0.14
occipital	cuneus	1.92 (0.12)	1.94 (0.13)	0.07	0.69	0.13	1.92 (0.14)	1.97 (0.15)	0.25	0.18	0.38
	lateraloccipital	2.15 (0.12)	2.15 (0.13)	-0.07	0.71	0.13	2.20 (0.14)	2.18 (0.17)	-0.23	0.22	0.35
	lingual	2.01 (0.12)	2.03 (0.13)	-0.01	0.98	0.12	2.04 (0.13)	2.06 (0.11)	0.05	0.77	0.13
	pericalcarine	1.83 (0.15)	1.84 (0.13)	0.06	0.71	0.13	1.82 (0.15)	1.82 (0.16)	-0.02	0.90	0.12
cingulate	caudalanteriorcingulate	2.62 (0.27)	2.65 (0.23)	-0.02	0.92	0.12	2.47 (0.30)	2.55 (0.21)	0.20	0.18	0.27
	isthmuscingulate	2.25 (0.36)	2.28 (0.34)	0.04	0.82	0.13	2.30 (0.21)	2.35 (0.18)	0.23	0.17	0.33
	posteriorcingulate	2.54 (0.19)	2.56 (0.18)	-0.07	0.70	0.13	2.51 (0.20)	2.58 (0.15)	0.27	0.09	0.46
	rostralanteriorcingulate	2.94 (0.32)	2.98 (0.24)	0.00	1.00	0.12	2.91 (0.31)	2.94 (0.25)	-0.02	0.90	0.12
Insula	insula	2.94 (0.23)	2.95 (0.16)	-0.08	0.62	0.14	2.96 (0.23)	2.99 (0.21)	-0.01	0.93	0.12

BF = Bayes Factor; Raw values are reported, statistics were done on normalized residuals

Table S3. Regional cortical surface area and subcortical volumes (mean (SD)).

		Left					Right				
lobe	Region (area/volume)	non-CUD	CUD	Cohen's *d*	*p*	BF	non-CUD	CUD	Cohen's *d*	*p*	BF
frontal	caudalmiddlefrontal	2177 (397)	2117 (383)	-0.21	0.24	0.29	1998 (423)	1937 (362)	-0.22	0.18	0.31
	frontalpole	197 (39)	187 (33)	-0.35	0.04	0.89	270 (54)	263 (46)	-0.15	0.39	0.19
	lateralorbitofrontal	2366 (286)	2351 (322)	-0.12	0.51	0.17	2358 (280)	2367 (309)	-0.05	0.78	0.13
	medialorbitofrontal	1777 (296)	1755 (307)	-0.08	0.65	0.14	1652 (209)	1642 (238)	-0.10	0.58	0.15
	paracentral	1264 (179)	1263 (209)	-0.04	0.84	0.13	1449 (209)	1426 (200)	-0.15	0.38	0.20
	parsopercularis	1522 (243)	1534 (245)	0.01	0.96	0.12	1296 (207)	1253 (220)	-0.24	0.19	0.37
	parsorbitalis	597 (88)	586 (94)	-0.18	0.33	0.23	744 (112)	735 (105)	-0.14	0.39	0.18
	parstriangularis	1264 (193)	1234 (175)	-0.23	0.17	0.33	1507 (243)	1432 (235)	-0.40	0.03	1.54
	precentral	4547 (534)	4537 (564)	-0.03	0.87	0.12	4658 (566)	4687 (595)	0.02	0.92	0.12
	rostralmiddlefrontal	5640 (784)	5624 (737)	-0.10	0.58	0.15	5894 (874)	5882 (777)	-0.10	0.53	0.15
	superiorfrontal	6760 (758)	6796 (816)	-0.02	0.93	0.12	6533 (804)	6637 (744)	0.09	0.59	0.15
medial temp	entorhinal	383 (69)	386 (88)	0.07	0.71	0.14	338 (69)	332 (68)	-0.05	0.77	0.13
	fusiform	3155 (413)	3193 (363)	0.05	0.76	0.13	3055 (420)	3083 (394)	0.03	0.87	0.12
	parahippocampal	686 (93)	723 (100)	0.34	0.06	0.87	675 (92)	678 (86)	-0.03	0.86	0.12
	temporalpole	481 (62)	478 (61)	-0.06	0.72	0.13	435 (62)	441 (69)	0.10	0.61	0.15
lateral temp	bankssts	1052 (180)	1045 (138)	-0.15	0.32	0.20	952 (157)	949 (170)	-0.10	0.60	0.15
	inferiortemporal	2990 (449)	3053 (461)	0.05	0.75	0.13	2872 (503)	2886 (456)	-0.05	0.77	0.13
	middletemporal	2876 (409)	2887 (461)	-0.06	0.73	0.13	3121 (425)	3147 (484)	-0.02	0.90	0.12
	superiortemporal	3660 (419)	3728 (473)	0.11	0.55	0.16	3489 (387)	3511 (406)	-0.03	0.88	0.12
	transversetemporal	445 (72)	446 (75)	-0.01	0.96	0.12	324 (55)	327 (60)	0.03	0.88	0.12
parietal	inferiorparietal	4499 (626)	4499 (618)	-0.09	0.62	0.14	5362 (770)	5372 (874)	-0.07	0.70	0.14
	postcentral	3906 (534)	4013 (498)	0.19	0.27	0.24	3766 (542)	3771 (520)	0.00	0.99	0.12
	precuneus	3464 (405)	3495 (466)	0.03	0.87	0.12	3675 (489)	3632 (475)	-0.14	0.41	0.18
	superiorparietal	5037 (542)	4993 (599)	-0.15	0.41	0.19	5020 (552)	4991 (681)	-0.07	0.70	0.14
	supramarginal	3497 (511)	3678 (589)	0.30	0.13	0.58	3437 (529)	3544 (528)	0.16	0.38	0.20
occipital	cuneus	1371 (233)	1332 (195)	-0.24	0.14	0.35	1405 (227)	1358 (247)	-0.23	0.20	0.32
	lateraloccipital	4540 (552)	4490 (559)	-0.17	0.34	0.21	4346 (620)	4513 (599)	0.25	0.16	0.37
	lingual	2871 (405)	2873 (399)	-0.01	0.94	0.12	2903 (399)	2889 (422)	-0.07	0.69	0.14
	pericalcarine	1370 (260)	1368 (218)	-0.03	0.84	0.12	1486 (287)	1447 (320)	-0.17	0.35	0.22
cingulate	caudalanteriorcingulate	624 (142)	601 (117)	-0.24	0.14	0.37	750 (180)	728 (121)	-0.16	0.31	0.20
	isthmuscingulate	1023 (359)	991 (307)	-0.12	0.46	0.16	892 (146)	884 (154)	-0.11	0.54	0.16
	posteriorcingulate	1114 (172)	1109 (145)	-0.07	0.67	0.14	1140 (180)	1114 (174)	-0.22	0.19	0.32
	rostralanteriorcingulate	748 (155)	775 (177)	0.14	0.45	0.18	597 (134)	624 (130)	0.22	0.21	0.30

insula	insula	2227 (256)	2233 (256)	0.00	0.99	0.12	2318 (286)	2325 (310)	0.03	0.86	0.12
subcortical	accumbens	538 (130)	578 (127)	0.18	0.30	0.24	550 (123)	607 (133)	0.37	0.04	1.18
	amygdala	1465 (236)	1509 (206)	0.10	0.53	0.15	1585 (248)	1570 (196)	-0.15	0.37	0.19
	caudate	3368 (540)	3372 (393)	-0.07	0.66	0.14	3518 (549)	3534 (444)	-0.05	0.77	0.13
	hippocampus	4249 (570)	4266 (476)	-0.09	0.56	0.15	4277 (504)	4215 (491)	-0.30	0.08	0.61
	pallidum	1282 (245)	1318 (258)	0.01	0.94	0.12	1394 (220)	1437 (205)	0.11	0.53	0.15
	putamen	5065 (869)	5152 (892)	-0.10	0.57	0.15	5010 (840)	5139 (771)	-0.02	0.91	0.12
	thalamus	7868 (1136)	7863 (945)	-0.08	0.65	0.14	6841 (955)	6860 (842)	-0.09	0.61	0.14
cerebellum	cerebellum	52945 (5681)	53047 (7516)	-0.09	0.66	0.14	54462 (6342)	54358 (7375)	-0.14	0.46	0.18

BF= Bayes Factor; Raw values are reported, statistics were done on normalized residuals

Table S4. Mean (SD) FA of the skeleton and of JHU white matter tracts.

	non-CUD	CUD	Cohen's *d*	*p*	BF
Whole skeleton	0.484 (0.015)	0.479 (0.015)	-0.34	0.057	0.86
L_ATR	0.498 (0.018)	0.492 (0.014)	-0.37	0.026 [1,2]	1.07
R_ATR	0.495 (0.017)	0.487 (0.015)	-0.44	0.014 [2]	2.19
L_CC	0.630 (0.025)	0.622 (0.024)	-0.34	0.060	0.80
R_CC	0.597 (0.028)	0.587 (0.026)	-0.34	0.054	0.84
L_CH	0.480 (0.030)	0.476 (0.032)	-0.14	0.455	0.19
R_CH	0.520 (0.030)	0.514 (0.032)	-0.20	0.292	0.26
L_CST	0.620 (0.018)	0.617 (0.021)	-0.13	0.505	0.18
R_CST	0.616 (0.018)	0.613 (0.020)	-0.14	0.467	0.18
Fmaj	0.592 (0.020)	0.584 (0.016)	-0.41	0.014 [1,2]	1.65
Fmin	0.556 (0.021)	0.552 (0.019)	-0.21	0.213	0.29
L_IFOF	0.527 (0.022)	0.519 (0.021)	-0.40	0.025 [1,2]	1.53
R_IFOF	0.534 (0.020)	0.527 (0.018)	-0.32	0.059	0.71
L_ILF	0.507 (0.022)	0.500 (0.021)	-0.34	0.056	0.85
R_ILF	0.513 (0.021)	0.507 (0.020)	-0.28	0.114	0.49
L_SLF	0.505 (0.018)	0.500 (0.019)	-0.31	0.102	0.60
R_SLF	0.517 (0.019)	0.510 (0.020)	-0.37	0.049 [1,2,3]	1.13
L_SLF_temp	0.541 (0.020)	0.535 (0.021)	-0.26	0.174	0.40
R_SLF_temp	0.544 (0.020)	0.537 (0.023)	-0.35	0.068	0.92
L_UF	0.476 (0.021)	0.470 (0.021)	-0.32	0.078	0.71
R_UF	0.477 (0.021)	0.473 (0.020)	-0.21	0.239	0.28

Underlined: $p < 0.05$, uncorrected
BF= Bayes Factor
ATR= Anterior Thalamic Radiation; CC= Cingulum Cortex; CH= Cingulum Hippocampus; CST= Corticospinal Tract; Fmaj= Forceps Major; FMin= Forceps Minor; IFOF= Inferior Fronto-Occipital Fasciculus; ILF= Inferior Longitudinal Fasciculus; SLF= Superior Longitudinal Fasciculus; SLF_temp= Superior Longitudinal Fasciculus Temporal Portion; UF= Uncinate Fasciculus; R= right; L=left.
Results were the same when not correcting for motion.

[1] not significant after controlling for nicotine dependence (FNDS)
[2] not significant after controlling for education; remains significant after controlling for social disability status
[3] not significant after controlling for alcohol use (drinks/month); remains significant when controlling for current diagnosis of alcohol use disorder

Table S5. Marginally significant results in regional graph-theoretical metrics. Significance was set at $p < 0.005$, uncorrected.

Metric	rs Module	Region	hemisphere	Cohen's *d*	*p*	BF*
Clustering (pos)	SC	Nucleus accumbens	Left	0.60	0.0022 [1,2]	11
Participation (neg)	DF	Superior frontal gyrus	Left	0.62	0.0015	15
Participation (neg)	VA	Insula	Left	0.55	0.0044 [3,4]	6.9
Participation (pos)	N	Middle Temporal Pole	Right	0.55	0.0038 [2,4,5]	8.0

BF= Bayes factor; FP= Fronto-Parietal; SC= subcortical; DF= Default mode; VA= ventral attention; N= None, regions in this module are noisy, results should be interpreted with caution.

* See supplemental text on interpretation of BF > 3 when doing multiple tests; this suggests that these BF provide strong evidence for the absence of a group difference.

[1] significant after correcting for nicotine dependence at $p < 0.0101$

[2] significant after correcting for education at $p < 0.0169$

[3] significant after correcting for alcoholic drinks/month or motion (DVARS) at $p < 0.0067$

[4] significant after correcting for social disability status at $p < 0.0077$

[5] significant after correcting for lifetime diagnosis of alcohol or substance use disorder other than cannabis, or motion (DVARS) at $p < 0.0070$

Table S6 – Marginally significant results in regional graph-theoretical metrics of non-GSR data. Significance was set at $p < 0.005$, uncorrected. Positive metrics were computed over densities 1-25%; negative metrics at 1-13% density.

Metric	rs Module	Region	hemisphere	Cohen's *d*	*p*	BF*
Positive metrics						
degree	CO	Insula	Left	0.59	0.0023	11.4
degree	DF	Angular gyrus	Right	0.57	0.0034	8.3
degree	VI	Middle temporal gyrus	Right	0.59	0.0024	10.8
strength	AU	Insula	Left	0.57	0.0029	9.0
strength	CO	Insula	Left	0.65	0.0008	24.6
strength	CO	Insula	Left	0.62	0.0013	15.8
strength	DF	Angular gyrus	Right	0.60	0.0015	12.9
strength	DA	Inferior parietal gyrus	Right	0.60	0.0016	12.5
strength	FP	Inferior parietal gyrus	Right	0.69	0.0005	47.4
strength	VI	Middle temporal gyrus	Right	0.61	0.0014	15.0
clustering	CO	Supplementary motor	Right	0.56	0.0036	7.2
clustering	DF	Precuneus	Right	0.58	0.0028	9.3
clustering	RT	Parahippocampal gyrus	Right	0.53	0.0050	5.4
clustering	CB	Vermis	-	0.55	0.0044	6.5
participation	DF	Superior frontal gyrus	Left	0.57	0.0036	8.4
participation	SA	Inferior frontal gyrus	Right	0.69	0.0004	42.1
Efficiency	-	Whole brain	-	0.42	0.0299	1.5
Negative metrics						
degree	CO	Supplementary motor	Right	0.57	0.0029	8.4
degree	N	Rectus	Right	0.60	0.0007	13.0
degree	N	Rectus	Right	0.59	0.0014	10.8
degree	VA	Insula	Left	-0.64	0.0010	22.7
strength	N	Rectus	Right	0.62	0.0014	16.2
strength	N	Rectus	Right	0.68	0.0004	40.9
strength	VA	Insula	Left	-0.70	0.0003	55.7
clustering	SC	Thalamus	Left	0.57	0.0031	8.1
clustering	SC	Caudate	Right	0.54	0.0047	5.7
participation	DA	Middle frontal gyrus	Left	0.58	0.0027	9.9
participation	N	Rectus	Right	0.60	0.0021	12.5
participation	RT	Parahippocampal gyrus	Right	0.66	0.0005	42.0

BF= Bayes factor; CO= cingulo-operculum ; DF= Default mode; VI= visual; AU= auditory; DA= dorsal attention; FP= Fronto-Parietal; RT= retrosplenial-temporal; CB= cerebellum; SA= salience; VA= ventral attention; SC= subcortical; N= None, regions in this module are noisy, results should be interpreted with caution.

* See supplemental text on interpretation of BF > 3 when doing multiple tests. This suggests BF < 35 provides substantial evidence for the absence of a group difference; BF >35 but < 300 are inconclusive.

Table S7 – Regional graph-theoretical metrics (*p* < 0.005) of GSR processed data with same density as non-GSR results: positive metrics were computed over densities 1-25%; negative metrics at 1-13% density.

Metric	rs Module	Region	hemisphere	Cohen's *d*	*p*	BF*
Positive metrics						
clustering	SC	Nucleus Accumbens	Left	0.62	0.0014	16.5
participation	N	Temporal pole	Right	0.59	0.0044	6.1
Negative metrics						
degree	DF	Superior temporal gyrus	Right	0.60	0.0017	12.0
degree	MM	Postcentral gyrus	Left	-0.57	0.0036	8.7
strength	DF	Superior temporal gyrus	Right	0.54	0.0045	5.8
participation	DF	Superior frontal gyrus	Left	0.56	0.0042	7.3
participation	N	Fusiform gyrus	Left	0.58	0.0028	9.7
participation	MH	Postcentral gyrus	Right	0.58	0.0023	9.8
participation	MH	Postcentral gyrus	Right	0.55	0.0041	6.9
participation	MH	Postcentral gyrus	Right	0.55	0.0048	6.3
participation	CB	Lobe V	Left	0.54	0.0047	6.1
participation	CB	Vermis, crus I	-	0.63	0.0010	18.5
participation	CB	Vermis, VIIIb	-	0.59	0.0022	11.1

BF= Bayes factor; SC= subcortical; DF= Default mode; MM= motor mouth area; MH= motor hand area; CB= cerebellum; N= None, regions in this module are noisy, results should be interpreted with caution.
* See supplemental text on interpretation of BF > 3 when doing multiple tests. This suggests BF < 19 provides substantial evidence for the absence of a group difference.

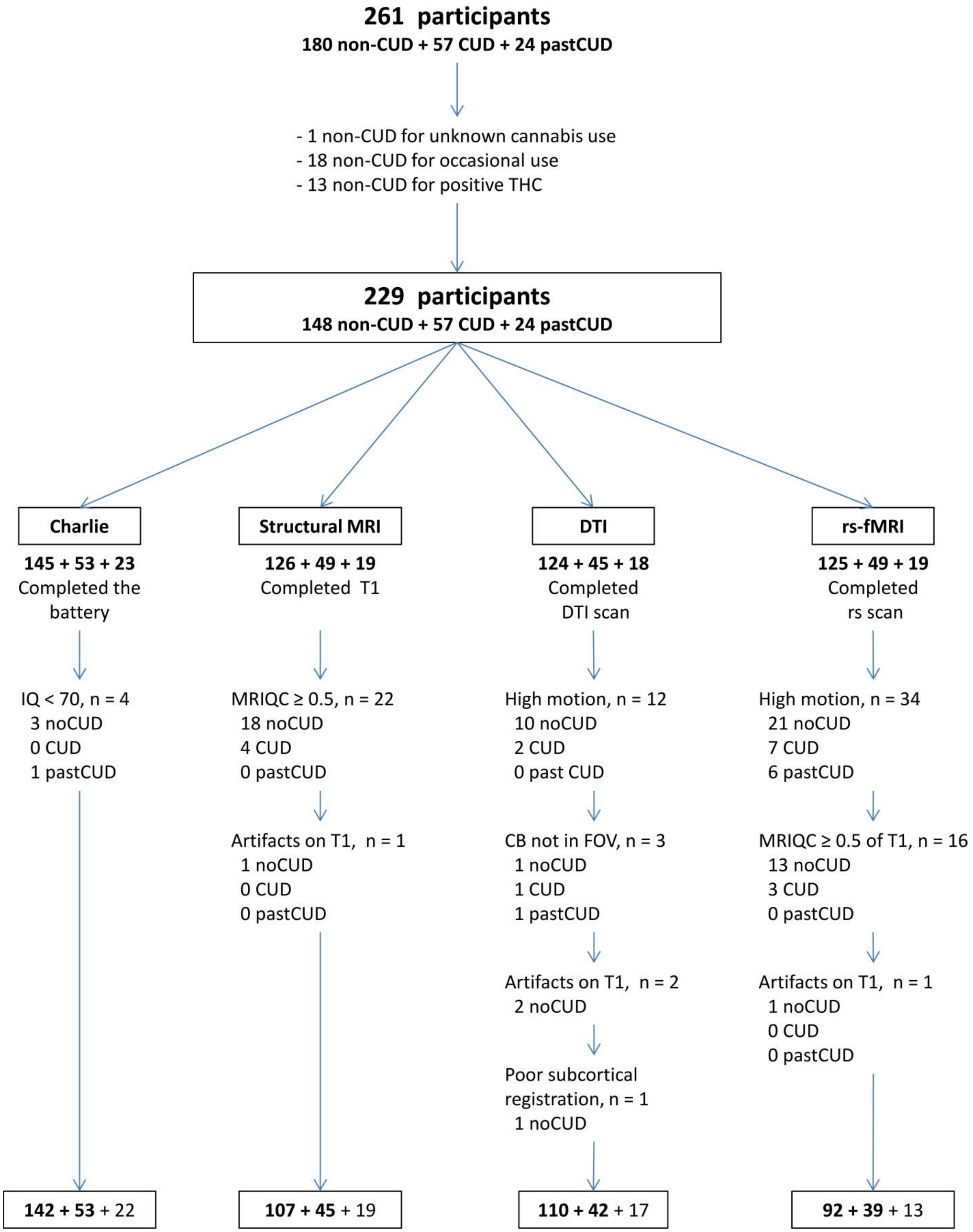

Figure S1. Overview of the number of participants per modality and reasons for exclusion. CB = cerebellum; rs = resting state.

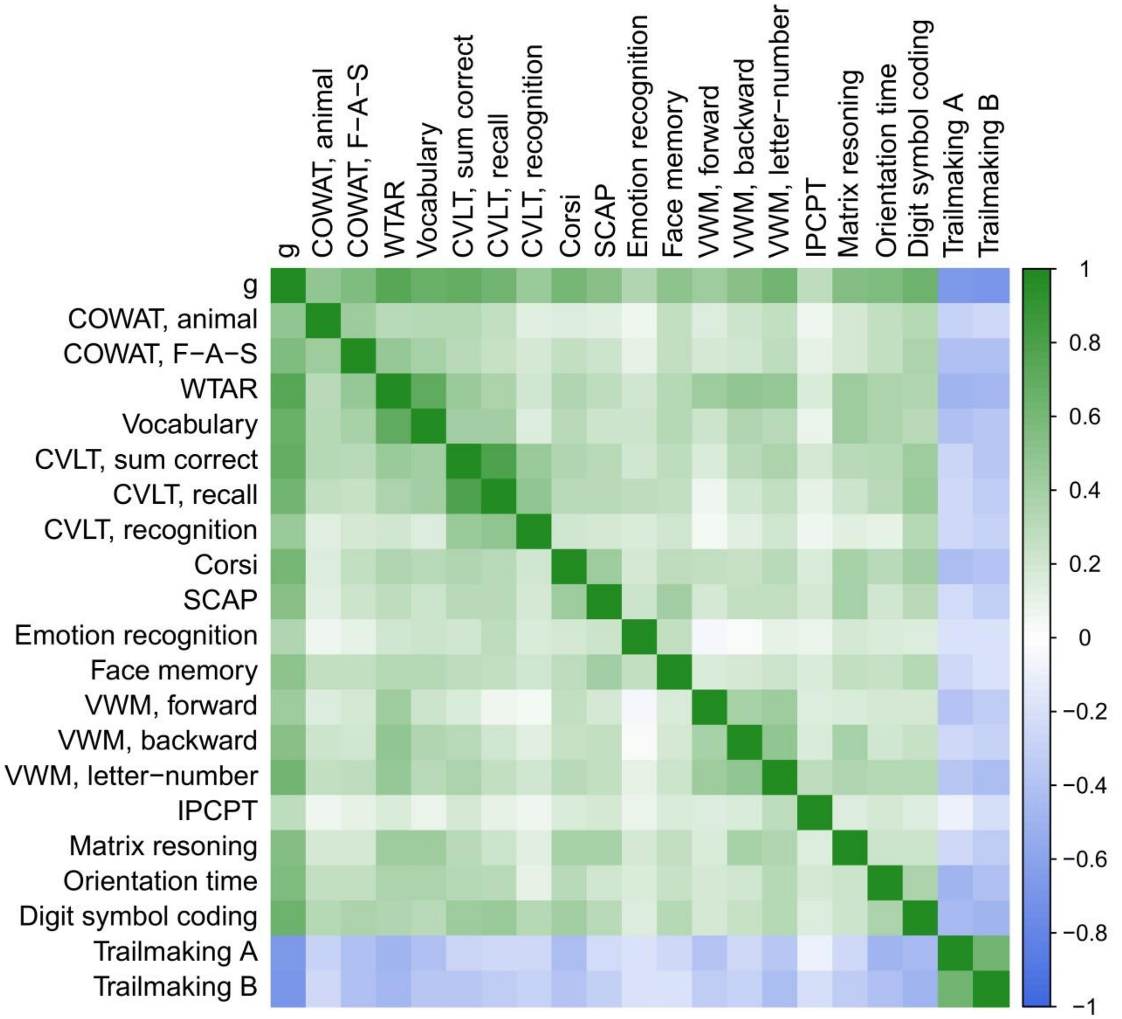

Figure S2. Correlations between all cognitive measures.

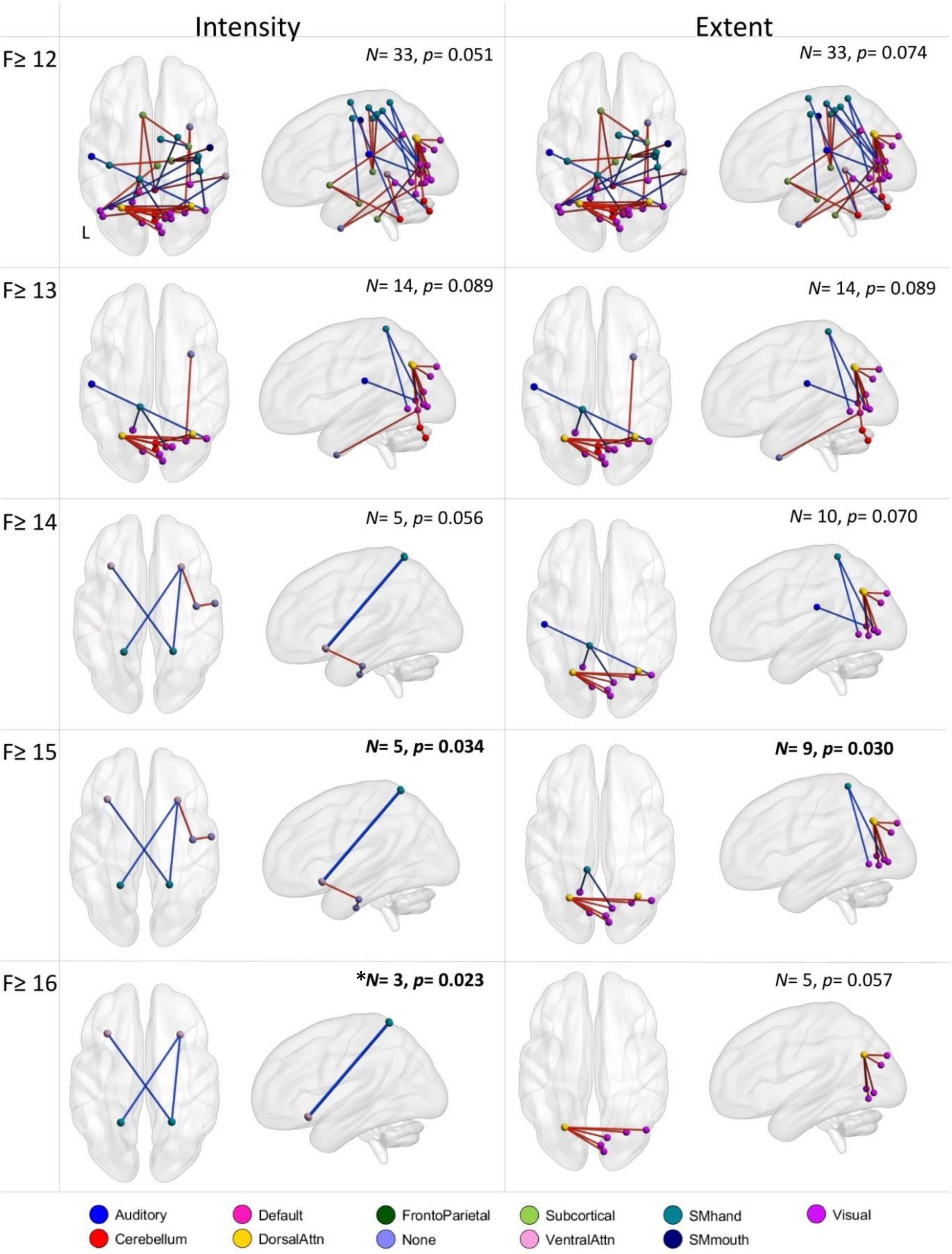

Figure S3. Output of all NBS networks over thresholds F≥12 to F≥16. *For intensity F≥17, the same network was found as for F≥16 (p=0.018). Blue edges indicate weaker FC in CUD, red edges indicate stronger FC in CUD. *N*= number of edges; SM= somato-motor cortex. Figures were created with BrainNet Viewer (Xia et al., 2013).

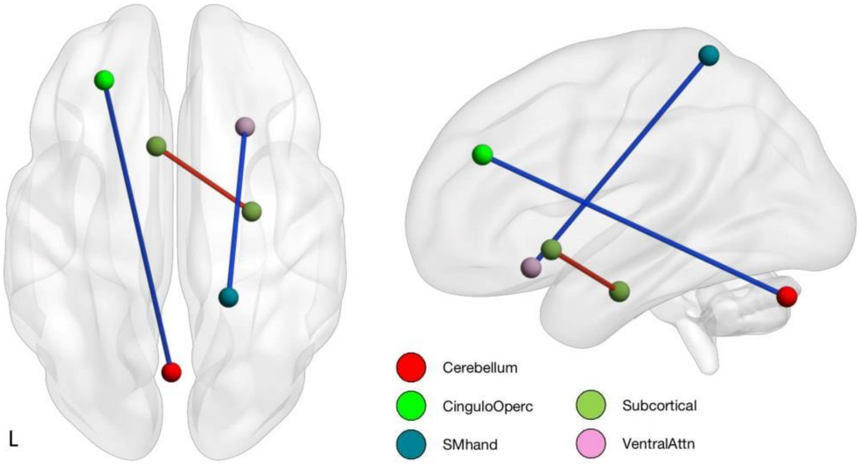

Figure S4. Three edges reached FDR significance: left middle frontal to vermis crus I; left nucleus accumbens to right hippocampus, right superior parietal gyrus to right insula. Note that for the FDR edges, the network is not thresholded in any way. Blue edges indicate weaker FC in CUD, red edges indicate stronger FC in CUD. SM= somato-motor cortex.

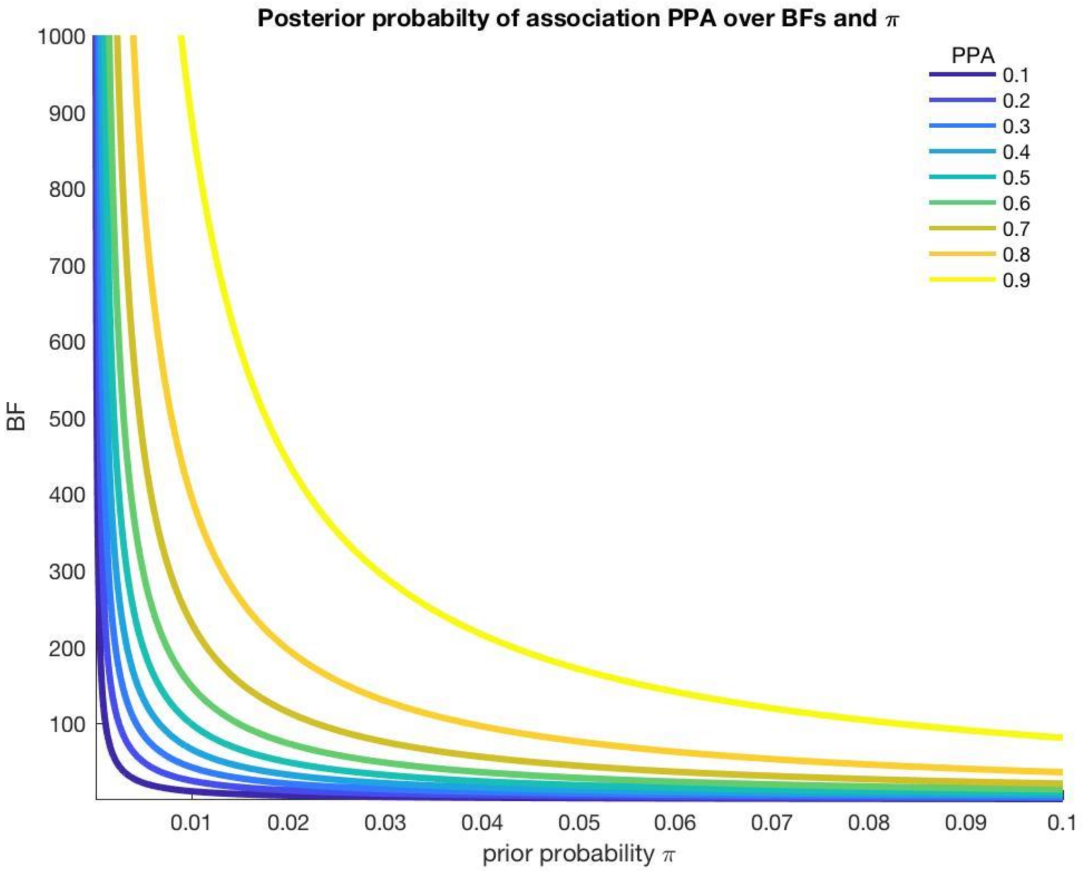

Figure S5. The relation between the prior probability π, the Bayes factor (BF) and the posterior probability of association PPA.
